# Genotype to ecotype in niche environments: adaptation of *Arthrobacter* to carbon availability and environmental conditions

**DOI:** 10.1038/s43705-022-00113-8

**Published:** 2022-03-30

**Authors:** Sara Gushgari-Doyle, Lauren M. Lui, Torben N. Nielsen, Xiaoqin Wu, Ria G. Malana, Andrew J. Hendrickson, Heloise Carion, Farris L. Poole, Michael W. W. Adams, Adam P. Arkin, Romy Chakraborty

**Affiliations:** 1 Lawrence Berkeley National Laboratory, Berkeley, CA USA; 2 Department of Biochemistry and Molecular Biology, University of Georgia, Athens, GA USA; 3 University of California, Berkeley, CA USA

**Keywords:** Biogeochemistry, Biogeochemistry

## Abstract

Niche environmental conditions influence both the structure and function of microbial communities and the cellular function of individual strains. The terrestrial subsurface is a dynamic and diverse environment that exhibits specific biogeochemical conditions associated with depth, resulting in distinct environmental niches. Here, we present the characterization of seven distinct strains belonging to the genus *Arthrobacter* isolated from varying depths of a single sediment core and associated groundwater from an adjacent well. We characterized genotype and phenotype of each isolate to connect specific cellular functions and metabolisms to ecotype. *Arthrobacter* isolates from each ecotype demonstrated functional and genomic capacities specific to their biogeochemical conditions of origin, including laboratory-demonstrated characterization of salinity tolerance and optimal pH, and genes for utilization of carbohydrates and other carbon substrates. Analysis of the *Arthrobacter* pangenome revealed that it is notably open with a volatile accessory genome compared to previous pangenome studies on other genera, suggesting a high potential for adaptability to environmental niches.

## Introduction

Environmental niches influence the structure and function of microbial communities [1, 2] as well as the genotype and phenotype of individual organisms, resulting in distinct ecotypes [3, 4]. Distinct ecotypes of a single species among widely varying environments (e.g., freshwater versus hot springs) have been well documented [5]. However, the influence of environmental heterogeneity at a small-scale spatial resolution (e.g., in a single sediment column and adjacent groundwater) on ecotypes of organisms phylogenetically considered to be the same genus are much less clear.

The terrestrial subsurface is an environment that commonly provides microniches that vary on a small spatial resolution. Geochemistry, carbon, and energy sources can vary radially and with depth in a terrestrial sediment [6], creating heterogeneity in physicochemical environments. The sediment zone at the interface of the water-saturated aquifer and the unsaturated vadose zone, often referred to as the variable saturated zone (VSZ), is a particularly dynamic environment offering a variety of niches. The VSZ undergoes steep gradients in hydraulic activity, geochemical conditions, and biological activity [7, 8] and is host to complex biogeochemical processes due to the interactions between solid, liquid, and gaseous environments [8]. It has been proposed that the VSZ is important for mass fluxes and nutrient cycling in terrestrial systems [9–11], and researchers have demonstrated that microbial community composition varies with depth in zones with fluctuating water tables [12]. However, much is unknown about functional differences of microbial communities in spatially adjacent, but diverse, environmental niches.

Because of the unique characteristics of the VSZ and adjacent zones, it is an ideal environment to investigate potential adaptation of closely related organisms to environmental niches. Several studies have linked environmental conditions to niche adaptation and specialization [13–15]. Mechanisms of horizontal gene transfer including acquisition of mobile elements [16] and plasmid-based uptake of genes [17] have both been implicated in the adaptive process of genomic evolution. Recently, pangenome analysis has also been used to suggest the importance of the genes present in accessory genomes of various genera in niche-specific adaptation [18]. These genomic signatures of adaptation, in addition to phenotype and environmental biogeochemistry, may lead to insights regarding the adaptation of microorganisms in the terrestrial subsurface.

Our study focuses on ecotypic differences of *Arthrobacter*, a highly prevalent subsurface bacterial genus, in the VSZ, the adjacent vadose and saturated zones, and the groundwater that interacts with the solid sediment phase. *Arthrobacter* is a diverse bacterial genus first described in 1947 [19]. It has been recovered from subsurface sediment environments ranging from deep mine clays [20] to karstic aquifers [21] and is also associated with rhizosphere and soil communities [22, 23]. *Arthrobacter* sp. are known to transform a wide range of organic carbon substrates, including aromatic hydrocarbons and various carbohydrates, and are therefore suspected to be key players in the transformation of subsurface carbon [24, 25]. Carbon substrates and metabolites have been identified as key drivers of microbial diversity [26, 27], and therefore may help elucidate differentiation of ecotypes among a single genus.

Here we present the isolation, genomic comparison, and laboratory characterization of seven *Arthrobacter* strains from groundwater and sediment samples collected from different depths of one sediment core and one groundwater well 1.5 m apart. We compare genes and pathways found in the isolates with physical-chemical attributes of the matrix from which they were isolated and experimentally demonstrated phenotypes to connect genotype and phenotype to ecotype, specifically focusing on carbon transformations. We then perform a pangenome analysis of *Arthrobacter* including the 41 publicly available circularized genomes from environmental origins to investigate carbon transformation and signatures of adaptation across the genus. Given the physicochemical heterogeneity of the terrestrial subsurface resulting in niche environments, we hypothesized that *Arthrobacter* would exhibit ecotypic diversity despite the close spatial origin of the isolates.

## Materials and methods

### Groundwater and sediment sampling

Groundwater and sediment samples were taken at the Oak Ridge Reservation Field Research Center (ORR FRC) in Oak Ridge, Tennessee, USA from wells FW305 and FW306, which are located 1.5 m apart at the pristine Background Area A at latitude and longitude (35.940969, –84.336186). The biogeochemistry of the ORR FRC has been described previously [28, 29].

### Geochemical and carbon characterization

Sediment-associated metals were extracted and measured using ICP-MS as previously described [4]. Dissolved organic matter was extracted according to the method previously developed in our lab [30]. Briefly, the sediment samples were freeze-dried and then extracted using Milli-Q water via rotary shaking overnight at 35 °C, followed by sonication in water bath for 2 h. The ratio of water to sediment was 4:1 (w/w). The extracts were centrifuged at 6000 *g* for 20 min. Then the supernatant was decanted and filtered through a polycarbonate filter (0.2 μm pore-sized, Whatman), followed by a second filtration step using a polyethersulfone filtration system (0.22 μm pore-sized, Corning). An aliquot of filtrate (15 mL) was freeze-dried, re-dissolved in 1 mL of methanol (HPLC grade, Fisher Scientific), and then filtered with polytetrafluoroethylene filter (0.2 μm pore-sized, Pall Corporation) prior to injection to the Fourier-transform ion cyclotron resonance mass spectrometry (FT-ICR MS) system. The FT-ICR MS instrumental analysis, molecular formulas assignment, and data processing were performed as described previously [30].

### Isolation

For groundwater isolates, 100 μL aliquots of groundwater from ORR FRC well FW305 was spread on 1/25 diluted R2A plates and incubated aerobically in the dark at 30 °C until the growth of colonies was observed. Colonies were picked from the spread plates and restreaked on 1/25 R2A medium until pure isolates were obtained. Isolates were grown in liquid 1/25 R2A medium until log-phase and cell pellets were collected for whole-genome sequencing. For sediment isolates, 1 g sediment was suspended in 100 mL RCH2 defined mineral medium to make a sediment slurry [31]. In all, 100 μL aliquots of liquid from sediment slurry were spread on different types of medium, including R2A, potato dextrose, modified Melin-Norkrans [32], and LB and incubated aerobically in the dark at 25 °C. Colonies were picked from the spread plates and restreaked on the respective medium until pure isolates were obtained. Isolates were grown in a liquid medium until log-phase and cell pellets were collected for whole-genome sequencing.

Isolate purity was confirmed via microscopy and 16S Sanger sequencing. All isolates were stored in glycerol medium at –80 °C for future experiments and were regrown from frozen glycerol stocks for each experiment.

### DNA extraction, whole-genome sequencing, and assembly

DNA extraction was carried out with two different methods so we could obtain high-quality short reads with Illumina sequencing and less accurate but longer reads with nanopore sequencing so we could use hybrid assembly to obtain circular, complete genomes. Nanopore reads can be used to span genomic repeats that prevent short read assemblies from producing circular genomes (see Supplementary Information X for more detail). DNA extraction kits that use silica columns, such as the Purelink Genomic DNA kit, often shear DNA and it is difficult to obtain enough high-molecular weight (HMW) DNA (20–50 kbp) that is desired for long read sequencing. In contrast, using HMW DNA for Illumina library prep can cause the library insert sizes to be too long for Illumina sequencing.

DNA for Illumina sequencing was extracted from cell pellets using the Purelink Genomic DNA Mini Kit (Thermofisher Scientific, USA) per manufacturer instructions. DNA quality and mass was quantified using Qubit 4 Fluorometer (Thermofisher Scientific, USA). DNA was sent to Novogene (California, USA) for whole-genome sequencing with 2 × 150 bp reads using Illumina Novaseq 6000 (Illumina, USA). Primer reads were trimmed by Novogene in-house.

HMW DNA was extracted for whole-genome sequencing using the NEB Monarch HMW DNA Extraction Kit for Tissue according to the manufacturer’s instructions with some modifications. For each isolate, 1 mL of culture at OD600 2.0 was pelleted. The pellet was digested with the following steps: resuspension in 300 μL PBS with 50 μL lysozyme (100 mg/mL) and incubated at 37 °C for 1 h, addition of 300 μL HMW gDNA Tissue Lysis Buffer and incubation at 500 rpm at 37 °C for 30 min using a Vortemp mixer, addition of 30 μL ProteinaseK and incubation for 30 min at 56 °C at 1400 rpm, and addition of 20 μL RNaseA and incubation for 10 min at 56 °C at 1400 rpm. The rest of the DNA extraction followed the manufacturer’s instructions. DNA mass was quantified using Qubit 4 Fluorometer (Thermofisher Scientific, USA) and the quality was assessed using Nanodrop (Thermofisher Scientific, USA). We confirmed the length of the HMW DNA on an agarose gel using the NEB Quick-Load 1 kb Extend DNA Ladder (Cat # N3239S).

For nanopore sequencing, DNA was barcoded using the Native Barcoding Expansion kit (Oxford Nanopore Technologies, UK), libraries were prepared using the SQK-LSK109 kit (Oxford Nanopore Technologies, UK) and sequenced on a MinION with an R9.4.1 flow cell. Illumina and nanopore reads were used as input to Unicycler for de novo hybrid assemblies. Illumina reads were cleaned and trimmed using BBtools (https://jgi.doe.gov/data-and-tools/bbtools) with default parameters. Adapter removal, barcode removal, and base calling were done on the nanopore reads using Guppy 4.0 (Oxford Nanopore Technologies, UK). Long read length distribution can be found in Supplementary Fig. S1. The Illumina and nanopore reads were assembled using Unicycler [33] with default parameters.

### Genome comparison

Once assembled, protein-coding regions and protein sequences were predicted with Prodigal (v2.6.3) [34] and genes were annotated with eggNOG-mapper [35]. Average amino acid identity (AAI) and orthologous fraction of genes were calculated using compareM (v0.1.1; https://github.com/dparks1134/CompareM). Digital DNA-DNA hybridization (dDDH) values were calculated using the DSMZ Genome-to-Genome Distance Calculator (v3.0; http://ggdc.dsmz.de/ggdc.php#) using BLAST+ as the alignment tool [36, 37]. Amino acid auxotrophy and small carbon compound catabolic capacity were evaluated via GapMind [38]. Carbohydrate-active enzymes (CAZy) were identified with dbCAN2 [39]. Genomic islands were identified with IslandViewer4 [40]. Plasmids were determined from the Unicycler assemblies, as the program indicates the length of the DNA elements and which ones are circular.

A phylogenetic tree of full-length 16S sequences was constructed of the seven isolates plus closest type-strain neighbors using IQ-TREE with default parameters and ultrafast bootstrap [41]. IQ-TREE selected GTR+F0 for the model. The 16S sequences were aligned with MAFFT [42] and manually trimmed to remove uninformative nucleotides (<5 nt) at the 3’ and 5’ ends using Jalview [43] (see Supplementary Files for the original and trimmed alignments). The tree was visualized with iTOL (v6.3.2) [44].

### Pangenome analysis

A pangenome comparing 34 publicly available *Arthrobacter* isolates and the seven isolates presented herein (for a total of 41 genomes) was assembled using Anvi’o (v6.2) [45] with NCBI blastp for amino acid sequence similarity search. All publicly available (NCBI and Joint Genome Institute databases), circularized genomes from unique organisms confirmed to belong to the genus *Arthrobacter* via comparison to the Ribosomal Database Project (v. RDP Taxonomy 18) [46] from the environmental origin as of September 2021 were included in pangenome analysis. Genes were categorized as part of the core genome (present in all genomes), soft-core genome (present in 95–99% of genomes), cloud genome (present in 1–5% of genomes), or shell genome (the remaining gene clusters). Pangenome openness was calculated using the equation *G* = *cN*^*γ*^ as previously described [47], where *G* is pangenome size, *c* is core genome size, *N* is the number of genomes included in the pangenome analysis, and γ is the openness coefficient.

### Phenotypic characterization

Several laboratory assays for phenotypic characterization were performed as described below, some of which were guided by results of the genomic comparison.

#### pH assay

To evaluate the effect of variable pH on isolate cell growth, isolates were grown aerobically in RCH2 defined mineral medium with pH ranging from 5 to 9 at 30 °C in the dark with shaking in biological triplicate. Citrate phosphate buffer was used at pH 5, phosphate buffer at pH 5.5–7.5, and tris-HCl buffer at pH 8–9. To measure cell growth, OD600 was measured every 3 h between 8:00 and 18:00 for 2 days.

#### Salinity assay

To evaluate the effect of increasing salinity on cell growth, isolates were grown aerobically in RCH2 defined mineral medium with additional concentrations of NaCl ranging from 0.5% to 10% (w/v) at 30 °C in the dark with shaking in biological triplicate. To measure cell growth, OD600 was measured every 3 h over 92 h. The IC50 for NaCl was calculated for each strain via linear interpolation.

#### Siderophore assay

Siderophore production was qualitatively evaluated using a colorimetric, overlay-chrome azurol S assay as previously described [48, 49]. A resulting purple color indicated catechol-type siderophore production, yellow indicated hydroxamate-type siderophore production, red-orange indicated a mix of different siderophore types, and blue indicated the absence of detected siderophore production.

#### Carbon source utilization assay

Each isolate was evaluated for cell growth with 58 different carbon sources (Supplementary Table S1). Isolates were grown aerobically in RCH2 basal medium with 10 mM carbon source (or 100 mg/L for complex carbons) at 30 °C in the dark with shaking in biological triplicate. To measure cell growth, OD_600_ was measured every 24 h for 4 days. Positive growth was determined by Student’s *t*-test with *p* < 0.05 of OD_600_ of each carbon source as compared to the negative control with inoculated cells but no amended carbon source.

#### Carbohydrate-active enzyme assay

To determine the activity of certain CAZy in each isolate, a CAZy assay was performed as previously described [50]. Briefly, carbohydrate hydrolytic enzymes and oxidative enzymes were screened in 96-well plates on liquid media containing one colorimetric detectable substrate. Cellulose-degrading enzymes cellulase, β-glucosidase, xylanase, and α-amylase were screened with AZCL-HE-Cellulose, PNP-β-D-Glucoside, AZCL-Xylan, and starch azure, respectively. Plates were incubated at 30 °C in the dark with shaking for 96 h, with measurements taken at 24 and 96 h. For measurement, cultures were centrifuged at 5000 g × 20 min and supernatant was decanted and measured at various wavelengths via microplate spectrophotometer (BioTek, VT, USA) for colorimetric detection. Substrate concentrations and detection wavelengths for colorimetric detection were performed as previously described [50].

### Statistical analyses

In quantitative laboratory assays, statistical significance (*p* < 0.05) of experimental conditions were evaluated against the control using ANOVA analysis in R. Statistically significant differences (*p* < 0.05) in the abundance of CAZymes of various types in each of the isolates presented herein were evaluated via one-way ANOVA analysis in R. Differences in the amounts of predicted CAZymes and simple carbon metabolisms by the environment of origin were also evaluated for statistical significance via one-way ANOVA analysis in R (*p* < 0.05). All ANOVA analyses in R were performed with default parameters (stats v. 3.6.2)

## Results

### Biogeochemical analysis and genome architecture

**Fig. 1:**
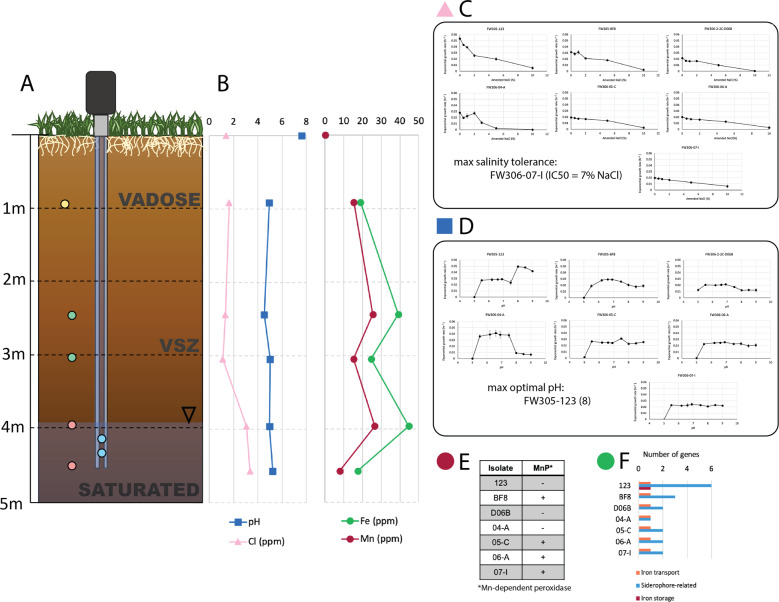
Sampling scheme and laboratory phenotyping results for *Arthrobacter* isolates. **A** Representation of the sampling location including depth below ground surface (BGS) and labels for the vadose zone, variable saturated zone (VSZ), and saturated zone. Markers indicate the locations of origin of each isolate. Isolates FW305-123 and FW-BF8 originated from the groundwater (blue), isolate FW306-2-2C-D06B from the vadose zone (yellow), isolates FW306-04-A and FW306-05-C from the VSZ (green), and both FW306-06-A and FW306-07-I from the saturated zone (pink). **B** pH, chloride (Cl), iron (Fe), and manganese (Mn) at varying depths BGS. Markers at 0 m BGS indicate groundwater measurements. Each marker represents the mean of analytical duplicates. **C** Plots representing exponential growth rate of each isolate with increasing salinity (NaCl). Larger versions of the plots can be found in Supplementary Fig. S6. Markers represent the mean of three biological replicates. Error bars indicate one standard deviation from the mean. **D** Plots representing exponential growth rate of each isolate with increasing pH. Larger versions of the plots can be found in Supplementary Fig. S4. Markers represent the mean of three biological replicates. Error bars indicate one standard deviation from the mean. **E** Presence (+) or absence (–) of Mn-dependent peroxidase in each isolate and **F** number of iron-related genes by categories of iron transport, siderophore-related, and iron storage present in the genome of each isolate.

Seven *Arthrobacter* strains were isolated and analyzed from the background zone of the ORR FRC in Oak Ridge, TN (Fig. 1A). In the sediment column, pH remained consistently between 4.5 ± 0.010 and 5.2 ± 0.070 from 1 to 5 m below ground surface (BGS), while the groundwater pH was 7.6 ± 0.10 (Fig. 1B). Extractable chloride was fairly constant between 0.91 m (1.64 ± 0.0919 ppm) to 3.0 m BGS (1.13 ± 0.0424 ppm) and increased in the saturated zone to a peak of 3.36 ± 1.11 ppm at 4.6 m. Total iron in the sediment was 19.0 ± 5.34 mg/kg at 0.91 m BGS and increased with depth, peaking at 44.8 ± 9.21 mg/kg at 4.0 m BGS (Fig. 1B). Total manganese followed a similar pattern with a measured sediment content of 15.36 ± 16.31 mg/kg at 0.91 m BGS and peaking at 26.6 ± 4.86 mg/kg at 4.0 m BGS. Groundwater contained low concentrations of total iron and manganese, with measured concentrations at 0.100 ± 0.0100 and 0.0500 ± 0.0100 ppm, respectively.

**Fig. 2:**
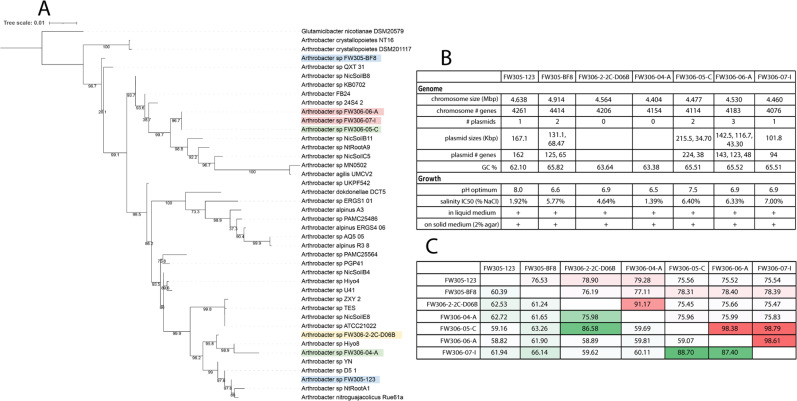
Phylogenetic tree and genomic and phenotypic summary of isolates. **A** Phylogenetic tree of the seven *Arthrobacter* isolates and other *Arthrobacter* with publicly available, complete genomes (SILVA r138.1). Label colors coordinate to those in Fig. 1A. **B** Genomic and phenotypic summary of the seven isolates. **C** Average amino acid identity (AAI, red) and orthologous fraction of genes (green) among the seven isolates. Color ranges correlate with values from low (white) to high (richest color).

Genomes were sequenced and reads assembled into circularized genomes, and a phylogenetic tree based on full-length 16S sequence was computed of the seven isolates and 34 publicly available *Arthrobacter* strains (Fig. 2A). Strains FW305-123 and FW305-BF8 were isolated from groundwater, FW306-2-2C-D06B from the vadose zone, FW306-04-A and FW306-05-C from the VSZ, and FW306-06-A and FW306-07-I from the saturated zone (Fig. 1A), The strains will be referenced without “FW305” and “FW306” prefixes henceforth. All isolate genomes exhibited between 4154 and 4604 protein-coding genes, with strain 04-A, isolated from the VSZ, possessing the smallest genome and BF8, a groundwater isolate, possessing the largest (Fig. 2B). To determine the whole-genome similarity between isolates with highly similar phylogenies, average AAI and orthologous gene fractions among fully circularized genomes were computed (Fig. 2C). dDDH was also performed to compare genomes (Supplementary Table S2). All isolates except vadose zone strain D06B and upper VSZ strain 04-A possessed at least one plasmid, with strains BF8 and 05-C possessing two and strain 06-A possessing three plasmids. All isolates with plasmids except saturated zone isolate 07-I exhibited multiple metals resistance genes encoded in their plasmids (Supplementary Fig. S2). Groundwater strains 123 and BF8, VSZ strain 05-C, and saturated zone strains 06-A and 07-I possessed plasmids containing carbon transformation metabolic genes including glycoside hydrolases, sugar isomerases, and alcohol dehydrogenases. The plasmid of VSZ strain 05-C also encoded a nitric oxide dioxygenase.

The seven isolate genomes contained several predicted genomic islands with groundwater strain 123 and deep sediment strain 07-I containing the fewest (7) and sediment strains 05-C and 06-A containing the most (14) (Supplementary Fig. S3). The genomic islands of these strains are largely dominated by antibiotic resistance, multidrug efflux, and various carbon metabolism genes (in addition to a large number of hypothetical proteins). While some genes residing in the genomic islands are shared among isolates (e.g., Fosfomycin resistance gene abaF in both groundwater isolates), others are unique. For example, genomic islands of groundwater strain BF8 contained molybdate-binding genes modAC and sediment strain 05-C contained several genes involved in lignin and plant cell wall degradation. A complete list of genes found in predicted genomic islands can be found in the Supplementary information (Supplementary Table S3).

### Genotypic and phenotyping correlation to geochemistry

Geochemical parameters are known to influence microbial function. Therefore, we investigated several geochemical parameters key to carbon transformations in VSZs in the terrestrial subsurface, including pH, salinity, and metals exhibiting variation by depth including iron and manganese.

#### pH

Structure and function of microbial communities in surface soil have been reported to vary with pH [51]. Vadose zone isolate D06B possessed the highest number of deaminases and decarboxylases, 11 and 15, respectively. Groundwater isolate BF8 possessed the second-highest number of these protein-coding genes with 10 of each. Isolate BF8 also possessed the highest number of sodium-proton antiporters at four. To further investigate pH tolerance in the isolates, a pH assay was performed. Groundwater isolates 123 and BF8 demonstrated optimal pH of 8.0 and 6.6, respectively. The sediment *Arthrobacter* exhibited wide pH ranges of relatively consistent growth rates (Fig. 1D and Supplementary Fig. S4). The optimal pH of vadose zone strain D06B was 6.9 and that of VSZ strains 04-A and 05-C were 6.5 and 7.5, respectively. Saturated zone strains 06-A and 07-I both exhibited optimal pH of 6.9.

#### Manganese

Manganese is critical for catalysis in several complex carbon-transforming enzymes, including Mn-dependent peroxidases and some glycoside hydrolasess [52, 53]. Deeper sediment isolates 05-C, 06-A, and 07-I exhibited higher numbers of glycoside hydrolases than the vadose zone isolate. Similarly, these isolates (in addition to groundwater isolate BF8) also possessed the Mn-dependent peroxidase (Fig. 1E).

#### Iron

Iron is an essential element for bacterial metabolism, but iron bioavailability varies among environmental matrices substantially. All isolates possessed the FTR1 family iron permease. The genomes of all sediment isolates encoded siderophore-related genes, and groundwater isolates 123 and BF8 possessed more siderophore-related genes that sediment isolates. However, none of the isolates exhibited production of siderophores in the phenotypic assay (Fig. 1F and Supplementary Fig. S5).

#### Variable salinity

Variable salinity is an environmental stressor associated with subsurface environments that undergo changes in water content [54]. It has been demonstrated that bacteria defend against salt stress, which is primarily osmotic stress, via two different mechanisms—KCl-type adaptation and compatible solute-type adaptation [55]. All isolates herein possess some genes for compatible solute-type osmoprotectant transport and synthesis, and the vadose zone isolate D06B possessed the highest number and highest diversity of osmoprotectant biosynthesis genes.

The isolates were evaluated for growth under increasing salinity concentrations (from 0.5% to 10% (w/v)), and the IC50 for NaCl was calculated for each strain (Fig. 1C and Supplementary Fig. S6). VSZ isolate 05-C and saturated zone isolate 07-I exhibited the highest salinity tolerances with IC50s of 6.40% and 7.00%, respectively. Groundwater strain 123 and VSZ strain 04-A exhibited the lowest salinity tolerances with IC50s of 1.92% and 1.39%, respectively. Strains BF8, D06B, and 06-A exhibited salinity IC50 values of 5.77%, 4.64%, and 6.33%, respectively.

#### Redox variability

While none of the sediment depths or groundwater contained high levels of nitrate (sediment NO_3_^–^ < 0.75 mg/kg, groundwater NO_3_^–^ = 0.34 ppm), saturated and VSZs are known to undergo redox fluctuations, sometimes incurring nitrate-reducing conditions [56]. VSZ (05-C) and saturated zone (06*-*A, 07-I) isolates all possess genes for dissimilatory nitrate reduction (narGHIJK).

### Simple carbon metabolism

The isolates were evaluated for the utilization of 58 different carbon sources associated with terrestrial sediment metabolisms (Supplementary Table S4). Groundwater strain 123 exhibited the fastest, most robust growth, reaching an OD_600_ of 0.98 ± 0.11 when grown in tryptic soy broth. Groundwater strain BF8 exhibited the slowest, least robust growth, reaching a maximum OD_600_ of 0.48 ± 0.32, also with tryptic soy broth as the carbon and energy source. Tryptic soy broth consistently yielded the most cellular growth for these isolates with the exception of strains 05-C and 06-A that exhibited the most robust growth utilizing folate (OD_600_ of 0.72 ± 0.55 and 0.69 ± 0.32, respectively). All sediment isolates except strain 04-A exhibited growth on polyols mannitol, ducitol, and myo-inositol, while strain 123 did not, and strain BF8 only exhibited growth utilizing mannitol. Saturated zone strain 07-I demonstrated growth on the largest number of substrates, 47.

Genomic capability of the seven isolates to catabolize 62 simple carbon substrates was evaluated. Strain 04-A demonstrated the lowest genomic capacity with 32 simple carbon substrates (with high or medium confidence) and strain 06-A demonstrated the highest genomic capacity with 37 substrates (Supplementary Table S5).

### Complex carbon metabolism

The relative fractions of complex carbon compounds in the groundwater and different sediment depths were determined via FT-ICR-MS. Sediment from 0 to 1 m BGS exhibited the highest proportion of carbohydrates at 21.2% (data not shown) and decreased as depth increased, representing only 0.5 and 1.3% of the carbon pool at 3–4 m BGS and 4–5 m BGS, respectively. In groundwater, carbohydrates accounted for only 0.6% of the carbon pool. Lignin compounds represented the highest fraction of complex carbon compounds (22.2%) in sediment from 3 to 4 m BGS.

**Fig. 3:**
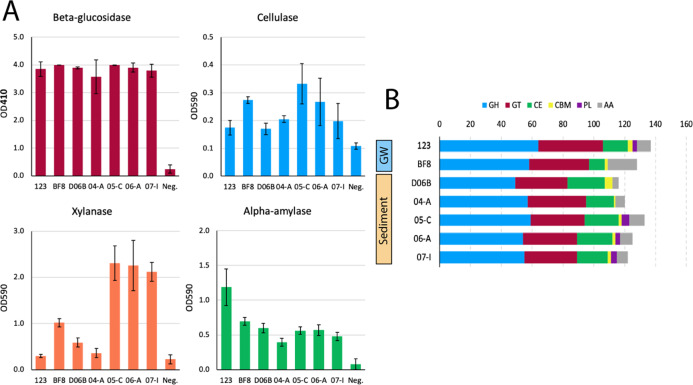
CAZy gene content and enzymatic assay results. **A** Activities of beta-glucosidase via OD410, and cellulase, xylanase, and alpha-amylase via OD590 for each of the seven isolates presented herein and a negative control. Error bars represent one standard deviation of biological triplicates. **B** Number of carbohydrate-active enzymes by CAZy database families glycoside hydrolase (GH), glycosyltransferase (GT), carbohydrate esterase (CE), carbohydrate-binding modules (CBM), polysaccharide lyase (PL), and auxiliary activity (AA). Isolates are grouped by the environment of origin: sediment and groundwater (GW).

Genomic comparison of CAZy genes was performed to connect the complex carbon transformation genotype to phenotype and ecotype (Fig. 3B). Of the seven isolates from this study, groundwater strain 123 possessed the highest number of CAZy with 137 and vadose zone strain D06B possessed the fewest with 116. However, all sediment strains possessed a greater number of carbohydrate esterases, with vadose zone strain D06B encoding the most with 24 and isolate 04-A from the upper VSZ encoding the least with 18. Groundwater isolates 123 and BF8 possessed 16 and 10 carbohydrate esterases, respectively.

Since all isolates possessed many glycoside hydrolase-coding genes (Fig. 3B), activities of several glycoside hydrolases were evaluated to determine functional differences in complex carbon transformation in the *Arthrobacter* isolates. All isolates exhibited similar activities of beta-glucosidase proteins, while isolates from the deeper sediment (05-C, 06-A, and 07-I) exhibited higher xylanase activity than the others (Fig. 3A). While all strains exhibited alpha-amylase activity, groundwater strain 123 exhibited the highest activity. All strains also exhibited cellulase activity to varying degrees (unpaired one-tail Student’s *t*-test, *p* < 0.05) with VSZ strain 05-C exhibiting the highest cellulase activity, followed by groundwater strain BF8 and saturated zone strain 06-A.

### Pangenome analysis

**Fig. 4:**
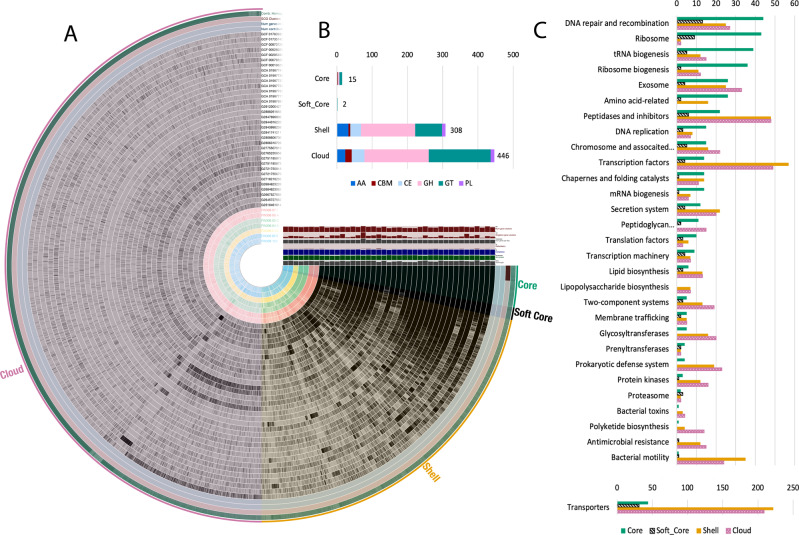
*Arthrobacter* pangenome analysis. **A** Visualized pangenome of the 41 *Arthrobacter* isolates with fully circularized genomes including core (present in all genomes), soft-core (present in 95–99% of genomes) shell (present in 5–95% of genomes), and cloud (present in 1–5% of genomes) gene clusters. **B** Number of carbohydrate-active enzyme protein-coding genes present in the core, soft-core, shell, and cloud genomes. **C** Gene clusters were categorized by KEGG Brite category and the fraction of gene clusters from each category contributing to each KEGG Brite module was plotted as a bar graph by core (green), soft-core (black stripe), shell (gold), and cloud (pink pattern). Gene clusters not mapping to a KEGG Brite module were not included in fraction calculation.

A pangenome containing the 34 publicly available *Arthrobacter* with circularized genomes [57–70] (Supplementary Table S6) and the seven genomes from this study was analyzed to determine the divergence of predicted capabilities within the *Arthrobacter* genus. Pangenome analysis estimated 28,182 unique gene clusters among the 41 analyzed genomes (Fig. 4) and revealed 986 core genes (common to all genomes), 294 soft-core genes (common to 95–99% of genomes), 8108 shell genes (common to 6–94% of genomes), and 18,794 cloud genes (common to 1–5% of genomes). Gene clusters were categorized by KEGG Brite category (Fig. 4A). Unsurprisingly, many of the core and soft-core genes included basic cellular function, cellular housekeeping, and central anabolism genes (e.g., amino acid biosynthesis) (Fig. 4C). One of the largest shell gene modules was for bacterial motility, which contained 35 gene clusters as compared to one in each of the core and soft-core pangenomes. Most of these 35 shell motility genes were flagellar biosynthesis proteins belonging to the fli and flg operons. Core and soft-core bacterial motility proteins were associated with pilus assembly. CAZy genes accounted for many gene clusters in the accessory genome, with 308 in the shell genome and 446 in the cloud (Fig. 4B).

CAZy were also evaluated in the individual 34 complete genomes included in the pangenome analysis. The total number of CAZymes varied widely from 52 to 172. Abundances of total CAZymes and of each variety were compared among the different environments of origin of the 41 analyzed genomes (Supplementary Fig. S7). Significant differences among environmental origins were observed in carbohydrate esterases at the *p* < 0.01 threshold (*p* = 0.00298) and glycosyltransferases at the *p* < 0.05 threshold (*p* = 0.0237) (one-way ANOVA).

## Discussion

Our results suggest that environmental factors are correlated with metabolic capabilities and cellular function in *Arthrobacter* isolated from different depths of a singular sampling location, demonstrating the adaptation of these bacteria to their environmental niches. The mostly closely related organisms were variable saturated and saturated zone strains 05-C, 06-A, and 07-I per AAI and orthologous fraction of genes. Although strains 123 and BF8 were both isolated from groundwater, they were very dissimilar, suggesting their initial environments of origin were also diverse.

Groundwater and the sediment vadose, VSZ, and saturated zones vary greatly in environmental conditions including water potential, salinity, pH, and redox. Unsurprisingly, the optimal pH of all strains was circumneutral with the exception of groundwater strain 123, which exhibited an optimal pH of 8.0, similar to the observed pH of the groundwater sample. More interestingly, all sediment strains exhibited relatively consistent growth rates over a large range of pH values from 5.5 to 9.0. While previous research has demonstrated microheterogeneity of pH in terrestrial subsurface environments [71], bacteria are generally known to exhibit narrow optimal pH ranges as compared to other microorganisms [51]. It is possible that this diversion from the expectation is due to the relatively higher number of studies on soil bacteria as compared to terrestrial sediment. Salinity was demonstrated to be highest in the sediment saturated zone and isolate 07-I exhibited the highest salinity tolerance, and the genomes of saturated zone strains 07-I and 06-A both possessed osmoprotectant-related genes in predicted genomic islands. This suggests that the saturated zone *Arthrobacter* have adapted to the higher salinity observed in the saturated zone. Genome analysis revealed differences in the nitrate-reducing potential of the seven strains. The three deeper sediment isolates exhibited the genomic capacity for nitrate reduction while the groundwater and vadose strains did not. While *Arthrobacter* have historically been regarded as obligate aerobic bacteria, some have demonstrated their capacity for denitrification in anaerobic conditions [22]. This is congruent with the environmental tenet that saturated sediments promote nitrate-reducing conditions [56], and that these organisms may have adapted to utilize alternative electron acceptors in variable redox conditions.

Abundances of iron and manganese, two metabolically important metals, are variable with depth and may influence the ecotypes of these *Arthrobacter*. The abundances of iron and manganese followed the same trend and were both higher in the deeper subsurface than in the vadose zone and groundwater. Groundwater isolates 123 and BF8 possessed the highest number of siderophore-related genes, which parallels the low concentration of iron found in the groundwater matrix, as these strains may be more frequently iron-starved and therefore require siderophores. No strains exhibited positive siderophore production results in the overlay-chrome azurol S assay. However, this may be due to assay sensitivity, the production of different siderophores, or specific siderophore production requirements by these strains. Manganese is an important cofactor in several complex carbon-transforming enzymes [52], including those found in *Arthrobacter* genomes [72]. Manganese has also been reported to increase the activity of complex carbon-transforming enzymes including beta-glucosidase [73]. The deeper sediment isolates 05-C, 06-A, and 07-I, originating from sediment zones exhibiting the highest manganese content, also possessed more glycoside hydrolase genes (some of which require manganese for catalysis [53]) than the vadose zone strain. In addition, these strains possessed Mn-dependent peroxidase genes supporting the potential reliance of metabolic processes on environmental conditions.

*Arthrobacter* are known for the transformation of complex carbon substrates [74]. All *Arthrobacter* isolates from this study possessed high numbers of CAZymes, with a minimum of 116 and groundwater isolate 123 possessing the highest number. The numbers of CAZymes present in previously reported *Arthrobacter* from other environments had a much wider range, with a minimum of 58 and a maximum of 148. The mean number of carbohydrate esterases in terrestrial sediment *Arthrobacter* was significantly greater (*p* < 0.1) than other environments, suggesting an augmented importance of carbohydrate metabolism in this part of the subsurface. The number of carbohydrate esterases encoded by each terrestrial sediment strain also followed a positive trend with the relative abundance of carbohydrates in their respective matrices, with the highest amount encoded in sediment vadose zone strain D06B. The differences in CAZyme gene content, which parallels the relative abundance of carbohydrates present in the environments of origin, suggest adaptation of the sediment *Arthrobacter* to the complex carbon substrates available in-situ as a function of depth. In addition, a large amount of total CAZy in groundwater strain 123 suggests this strain has augmented its genome to be able to metabolize a high diversity of substrates. Similarly, a notable decrease in carbohydrate transport and metabolism genes has been observed in Antarctic *Arthrobacter*, parallel to the availability of substrate and relative cost of metabolic versatility in the harsh Antarctic soil environment [75]. Many of the results presented herein demonstrate the stark differences between the two groundwater strains. Depending on geology and aquifer structure, groundwater can transport microorganisms at rapid velocities [76]. Therefore, these groundwater *Arthrobacter* are likely transient as compared to their sediment-dwelling counterparts; they could have originated from anywhere.

Pangenome analysis revealed that the genus *Arthrobacter* has a notably open pangenome with an openness coefficient close to one (*γ* = 0.903). Previous studies have suggested a link between open pangenomes and environmental adaptation as a driver of evolution [77–79]. In addition, in support of this, gene clusters present in the *Arthrobacter* shell and cloud gene cluster groups, but less so in the core and soft-core groups, were largely related to environment-specific processes such as CAZymes and two-component systems for environmental sensing. Moreover, transporters were also predominantly present in the shell and cloud genomes as compared to the core and soft-core, suggesting high variation in transporter specificity among closely related organisms from different environments.

In addition to the gene presence and phenotypic evidence for adaptation of *Arthrobacter* to environmental niches and carbon availability discussed above, we can also look to the genomic architecture of genes related to these particular functions. Plasmids, genomic islands, and other mobile elements are known to distribute genes that provide selective advantages to the host organisms to survive in different environmental niches [80]. Groundwater bacteria have been shown to possess large plasmids with many metal resistance genes [81]. Both groundwater isolates evaluated herein and sediment strains 05-C and 06-A adhered to this observation. However, the isolate from the deepest sediment zone, strain 07-I, did not encode any metal resistance genes, but rather encoded a variety of metabolic genes including sugar isomerases, alcohol dehydrogenases, and genes related to binding and transport of carbon substrates. Genes present in genomic islands can also help elucidate the functional adaptation of organisms to their environments [82]. All of the isolates demonstrated the presence of genes for various carbon degradation pathways in genomic islands. Notably, sediment isolate 05-C, originating from sediment between 3 and 4 m BGS that contained the highest fraction of lignin compounds, possessed genomic islands with genes related to lignin and plant cell wall degradation. Other isolates exhibited unique genes related to carbohydrate and aromatic compound degradation. To connect these specific functions to environmental niches, further research is needed to more deeply characterize the variation in complex carbon structure with depth. The plasmids and genomic islands of each isolate encoded unique signatures of genes providing important functions for their relative environments of origin, supporting the importance of plasmid plasticity in adaptation to unique and variable environments.

The high degree of openness of the *Arthrobacter* pangenome as compared to those of other genera (e.g., *Janthinobacterium* [83], *Pectobacterium* [84], *Yersinia* [85]), as well as the genes present in genomic islands and plasmids, suggests differences in adaptability among different taxa. As some microorganisms compete in communities strategically by leveraging catalysis kinetics or substrate affinities, it has been suggested that others may avoid direct competition via substrate specialization [26]. To achieve substrate specialization, *Arthrobacter* and other genera may demonstrate higher capacities for genome editing to adapt to diverse and changing environments. Non-environmental *Arthrobacter* have also been suggested to incorporate genes into their genome for performing habitat-specific functions, such as in the beetle host-associated *Arthrobacter ipsi* [86]. As we continue to find *Arthrobacter* in highly diverse environments, the genus may be a suitable model organism to study rapid adaptation to environmental niches. Further research is necessary to continue exploring the propensity of certain genera to demonstrate this activity. It is also possible that the notably high openness coefficient may be due to the current standards in taxonomic classification, which still largely utilize 16S rRNA, especially in curated databases [46, 87]. While the results of the pangenome analysis suggest that *Arthrobacter* are highly adaptive to their environment, they also incite the discussion of how the scientific community delineates taxa (i.e., should such vast metabolic diversity be considered a single genus). This discussion will require substantially more data, analysis, and discourse within the scientific community and has the potential for a significant impact on microbial ecology.

The results of this study suggest the adaptation of members of a single genus at a single terrestrial sampling location to the ecological niches provided by varying depth, supporting previous studies reporting the occurrence of microbial evolution to allow for rapid adaptation to environmental conditions [82, 88, 89]. Isolates demonstrated both genotypic and phenotypic characteristics suited for groundwater, the vadose zone, the VSZ or the saturated zone, allowing us to draw connections among genotype, phenotype, and ecotype. These results also highlight the variability of metabolism and cellular functions in members of the genus *Arthrobacter*; an important consideration when identifying “representative” or “model” organisms for ecological study. Further research is required to elucidate the mechanisms behind genome reduction or expansion due to adaptation to a niche environment.

## Supplementary information

Supplementary File

Supplementary Data2

Supplementary Data3

Supplementary Data4

## Data Availability

The genomes sequenced and analyzed for this study are available in the NCBI repository under BioProject PRJNA766658, BioSample accession numbers SAMN21856737 – 21856743.
