# Supplementary material for: Genotype to ecotype in niche environments: adaptation of Arthrobacter to carbon availability and environmental conditions
Source: ISME Commun. 2022 Mar 30;2:32. doi: 10.1038/s43705-022-00113-8 (PMC9723602; doi:10.1038/s43705-022-00113-8)
Supplement: Supplementary file 1 — Supplementary File [file 43705_2022_113_MOESM1_ESM.pdf]

Supplemental Information

Title: Genotype to ecotype in niche environments: Adaptation of *Arthrobacter* to carbon availability and environmental conditions.

Running title: Genotype-to-ecotype comparison of *Arthrobacter* strains

Authors: Sara Gushgari-Doyle,[1] Lauren M. Lui,[1] Torben N. Nielsen,[1] Xiaoqin Wu,[1] Ria G. Malana,[1] Andrew J. Hendrickson[1], Heloise Carion[1], Farris L. Poole II,[2] Michael W.W. Adams,[2] Adam P. Arkin,[1,3] and Romy Chakraborty.[1]

Affiliations: [1]Lawrence Berkeley National Laboratory, Berkeley, CA; [2]Department of Biochemistry and Molecular Biology, University of Georgia, Athens, GA; [3]University of California, Berkeley, CA.

Supplemental information on the use of Unicycler for assembling complete genomes

To generate a finished genome, it is necessary to have reads that are longer than the longest repeat. This precludes the use of only Illumina reads as they are typically 2 x 150 bp from the ends of a fragment that is approximately 500 bp long since most microbes we deal with have repeats that are longer than 500 bp. The ribosomal operon is a case in point; for most organisms with a copy number greater than one, it introduces a repeat of length ~7,000 bp. In order to generate a finished genome, we employed Oxford Nanopore Technologies (ONT) sequencing and got reads of sufficient length to span the repeats (see Supplementary Figure S1 for the long read length distributions of each sample).

Unicycler (Wick et al., 2017) generates an assembly by using the SPAdes assembler with varying kmer values to generate multiple short read assemblies. Out of these, Unicycler picks the one that reduces the complexity of the assembly graph the most. The short read assembly is accurate in terms of base calling errors, but fragmented. In the second phase, Unicycler uses the (less accurate) long reads to join the short reads together and resolve repeats where possible. Finally, Unicycler makes a determination as to which of the long pieces are circular (find long reads that span the contig ends) and it uses the short Illumina reads in a final polishing pass in an attempt to eliminate remaining errors and misassemblies using Pilon (Walker et al., 2014). Unicycler will also attempt to find a start gene on the circular pieces and if successful, it will rotate the piece to start with that gene (dnaA is typically used).

Generally, plasmids or phage show up as circular pieces that are significant smaller than the main chromosome and also lack ribosomal RNA.

Unicycler is a “short read first assembler” and given high Illumina coverage along with moderate nanopore read coverage, is able to produce finished genomes. There are also long read first assemblers. Flye (Kolmogorov et al., 2019) is the most popular of these that we are aware of and we commonly use it as a check on the Unicycler assemblies we generate. In most cases, the resulting assemblies appear equivalent and this provides a check on assembly quality.

References:
Kolmogorov, M., Yuan, J., Lin, Y., & Pevzner, P. A. (2019). Assembly of long, error-prone reads using repeat graphs. *Nature Biotechnology*, *37*(5), 540–546. https://doi.org/10.1038/s41587-019-0072-8

Walker, B. J., Abeel, T., Shea, T., Priest, M., Abouelliel, A., Sakthikumar, S., Cuomo, C. A., Zeng, Q., Wortman, J., & Young, S. K. (2014). Pilon: an integrated tool for comprehensive microbial variant detection and genome assembly improvement. *PloS One*, *9*(11), e112963.

Wick, R. R., Judd, L. M., Gorrie, C. L., & Holt, K. E. (2017). Unicycler: resolving bacterial genome assemblies from short and long sequencing reads. *PLoS Computational Biology*, *13*(6), e1005595.

Table S1. List of 58 carbon sources included in the carbon source utilization assay.

cytosine
thymidine
inosine
cytodine
uridine
adenosine
adenine
tryptophan
arginine
asparagine
aspartate
glutamate
histidine
glutamine
cysteine
tyrosine
serine
proline
isoleucine
methionine
phenylalanine
citrulline
glucosamine
casamino acids
xylitol
mannitol
ducitol
myo-inositol
acetate
malate
folate
succinate
propionate
formate
butyrate
fumarate
citrate
lactate
pyruvate
glycolic acid
nicotinic acid
pthalate
sucrose
glucose
lactose
ribose
mannose
fructose
rhamnose
maltose
arabinose
cellobiose
cellulose
glucuronamide
urea
tryptone
R2A
TSB

Table S2. Digital DNA-DNA Hybridization analysis on the seven isolate genomes.

Query genome: FW305-123

	**Formula 1**				**Formula 2**				**Formula 3**				**G+C difference**
Reference genome	DDH	Model C.I.	Distance	Prob. DDH >= 70%	DDH	Model C.I.	Distance	Prob. DDH >= 70%	DDH	Model C.I.	Distance	Prob. DDH >= 70%	
FW305-123	100	[100 - NaN%]	0	99.62	100	[100 - NaN%]	0	98.3	100	[100 - NaN%]	0	99.99	0
FW306-07-I	18.2	[15.1 - 21.7%	0.7648	0	20.9	[18.6 - 23.3%	0.2105	0	17.8	[15.2 - 20.8%	0.8143	0	3.49
FW305-BF8	18.1	[15 - 21.6%]	0.7673	0	21.3	[19.1 - 23.7%	0.2059	0	17.8	[15.1 - 20.8%	0.8153	0	3.8
FW306-2-2C-D06B	21	[17.8 - 24.6%	0.6861	0	20.9	[18.7 - 23.4%	0.2098	0	20.1	[17.3 - 23.1%	0.752	0	1.53
FW306-04-A	20	[16.8 - 23.6%	0.7114	0	21.2	[19 - 23.6%]	0.2071	0	19.3	[16.6 - 22.4%	0.7712	0	1.28
FW306-05-C	18.1	[15 - 21.6%]	0.7673	0	20.8	[18.6 - 23.3%	0.2108	0	17.7	[15.1 - 20.7%	0.8164	0	3.53
FW306-06-A	17.8	[14.7 - 21.3%	0.778	0	20.9	[18.7 - 23.3%	0.2103	0	17.5	[14.9 - 20.4%	0.8247	0	3.57

Query genome: FW305-BF8

	**Formula 1**				**Formula 2**				**Formula 3**				**G+C difference**
Reference genome	DDH	Model C.I.	Distance	Prob. DDH >= 70%	DDH	Model C.I.	Distance	Prob. DDH >= 70%	DDH	Model C.I.	Distance	Prob. DDH >= 70%	
FW305-123	18.1	[15 - 21.6%]	0.7673	0	21.3	[19.1 - 23.7%	0.2059	0	17.8	[15.1 - 20.8%	0.8153	0	3.8
FW306-07-I	23.8	[20.5 - 27.4%	0.6227	0.01	22.6	[20.3 - 25%]	0.1941	0	22.5	[19.7 - 25.6%	0.6959	0	0.31
FW305-BF8	100	[100 - NaN%]	0	99.62	100	[100 - NaN%]	0	98.3	100	[100 - NaN%]	0	99.99	0
FW306-2-2C-D06B	19	[15.8 - 22.6%	0.7404	0	22.1	[19.8 - 24.5%	0.1988	0	18.6	[15.9 - 21.6%	0.7921	0	2.27
FW306-04-A	18.8	[15.7 - 22.4%	0.7441	0	21.7	[19.5 - 24.1%	0.2021	0	18.4	[15.8 - 21.5%	0.7959	0	2.52
FW306-05-C	23.8	[20.5 - 27.5%	0.6211	0.01	22.5	[20.2 - 24.9%	0.1952	0	22.5	[19.7 - 25.6%	0.695	0	0.27
FW306-06-A	23.7	[20.4 - 27.3%	0.6243	0.01	22.6	[20.3 - 25%]	0.194	0	22.4	[19.6 - 25.5%	0.6971	0	0.23

Query genome: FW306-2-2C-D06B

	**Formula 1**				**Formula 2**				**Formula 3**				**G+C difference**
Reference genome	DDH	Model C.I.	Distance	Prob. DDH >= 70%	DDH	Model C.I.	Distance	Prob. DDH >= 70%	DDH	Model C.I.	Distance	Prob. DDH >= 70%	
FW305-123	21	[17.8 - 24.6%]	0.6861	0	20.9	[18.7 - 23.4%	0.2098	0	20.1	[17.3 - 23.1%]	0.752	0	1.53
FW306-07-I	18.2	[15.1 - 21.8%]	0.7633	0	21.5	[19.3 - 24%]	0.2037	0	17.9	[15.3 - 20.9%]	0.8115	0	1.96
FW305-BF8	19	[15.8 - 22.6%]	0.7404	0	22.1	[19.8 - 24.5%	0.1988	0	18.6	[15.9 - 21.6%]	0.7921	0	2.27
FW306-2-2C-D06B	100	[100 - NaN%]	0	99.62	100	[100 - NaN%]	0	98.3	100	[100 - NaN%]	0	99.99	0
FW306-04-A	56.7	[53.1 - 60.2%]	0.2606	30.39	33.8	[31.4 - 36.4%	0.1232	0.45	50.8	[47.7 - 53.8%]	0.3517	3.43	0.26
FW306-05-C	18.3	[15.2 - 21.9%]	0.7608	0	21.6	[19.4 - 24.1%	0.2029	0	18	[15.3 - 21%]	0.8093	0	2
FW306-06-A	18.2	[15.1 - 21.7%]	0.7649	0	21.7	[19.4 - 24.1%	0.2022	0	17.9	[15.2 - 20.9%]	0.8125	0	2.04

Query genome: FW306-04-A

	Formula 1				Formula 2				Formula 3				G+C difference
Reference genome	DDH	Model C.I.	Distance	Prob. DDH >= 70%	DDH	Model C.I.	Distance	Prob. DDH >= 70%	DDH	Model C.I.	Distance	Prob. DDH >= 70%	
FW305-123	20	[16.8 - 23.6%	0.7114	0	21.2	[19 - 23.6%]	0.2071	0	19.3	[16.6 - 22.4%]	0.7712	0	1.28
FW306-07-I	18.1	[15.1 - 21.7%	0.7655	0	21.5	[19.2 - 23.9%]	0.2045	0	17.8	[15.2 - 20.8%]	0.8135	0	2.21
FW305-BF8	18.8	[15.7 - 22.4%	0.7441	0	21.7	[19.5 - 24.1%]	0.2021	0	18.4	[15.8 - 21.5%]	0.7959	0	2.52
FW306-2-2C-D06B	56.7	[53.1 - 60.2%	0.2606	30.39	33.8	[31.4 - 36.4%]	0.1232	0.45	50.8	[47.7 - 53.8%]	0.3517	3.43	0.26
FW306-04-A	100	[100 - NaN%]	0	99.62	100	[100 - NaN%]	0	98.3	100	[100 - NaN%]	0	99.99	0
FW306-05-C	18.1	[15 - 21.6%]	0.7682	0	21.5	[19.3 - 23.9%]	0.2041	0	17.8	[15.1 - 20.8%]	0.8155	0	2.25
FW306-06-A	18.1	[15 - 21.6%]	0.7679	0	21.4	[19.2 - 23.8%]	0.205	0	17.8	[15.1 - 20.8%]	0.8155	0	2.29

Query genome: FW306-05-C

	Formula 1				Formula 2				Formula 3				G+C difference
Reference genome	DDH	Model C.I.	Distance	Prob. DDH >= 70%	DDH	Model C.I.	Distance	Prob. DDH >= 70%	DDH	Model C.I.	Distance	Prob. DDH >= 70%	
FW305-123	18.1	[15 - 21.6%]	0.7673	0	20.8	[18.6 - 23.3%]	0.2108	0	17.7	[15.1 - 20.7%]	0.8164	0	3.53
FW306-07-I	90.5	[87.3 - 92.9%	0.0782	97.46	90	[87.6 - 91.9%]	0.0122	95.78	92.9	[90.6 - 94.7%]	0.0894	99.71	0.04
FW305-BF8	23.8	[20.5 - 27.5%	0.6211	0.01	22.5	[20.2 - 24.9%]	0.1952	0	22.5	[19.7 - 25.6%]	0.695	0	0.27
FW306-2-2C-D06B	18.3	[15.2 - 21.9%	0.7608	0	21.6	[19.4 - 24.1%]	0.2029	0	18	[15.3 - 21%]	0.8093	0	2
FW306-04-A	18.1	[15 - 21.6%]	0.7682	0	21.5	[19.3 - 23.9%]	0.2041	0	17.8	[15.1 - 20.8%]	0.8155	0	2.25
FW306-05-C	100	[100 - NaN%]	0	99.62	100	[100 - NaN%]	0	98.3	100	[100 - NaN%]	0	99.99	0
FW306-06-A	87.3	[83.7 - 90.1%	0.0955	96.16	87.5	[84.9 - 89.7%]	0.0149	94.86	90.1	[87.3 - 92.3%]	0.109	99.43	0.04

Query genome: FW306-06-A

	Formula 1				Formula 2				Formula 3				G+C difference
Reference genome	DDH	Model C.I.	Distance	Prob. DDH >= 70%	DDH	Model C.I.	Distance	Prob. DDH >= 70%	DDH	Model C.I.	Distance	Prob. DDH >= 70%	
FW305-123	17.8	[14.7 - 21.3%	0.778	0	20.9	[18.7 - 23.3%	0.2103	0	17.5	[14.9 - 20.4%]	0.8247	0	3.57
FW306-07-I	87.9	[84.5 - 90.7%	0.0919	96.47	87.9	[85.4 - 90%]	0.0144	95.02	90.7	[88 - 92.8%]	0.105	99.5	0.08
FW305-BF8	23.7	[20.4 - 27.3%	0.6243	0.01	22.6	[20.3 - 25%]	0.194	0	22.4	[19.6 - 25.5%]	0.6971	0	0.23
FW306-2-2C-D06B	18.2	[15.1 - 21.7%	0.7649	0	21.7	[19.4 - 24.1%	0.2022	0	17.9	[15.2 - 20.9%]	0.8125	0	2.04
FW306-04-A	18.1	[15 - 21.6%]	0.7679	0	21.4	[19.2 - 23.8%	0.205	0	17.8	[15.1 - 20.8%]	0.8155	0	2.29
FW306-05-C	87.3	[83.7 - 90.1%	0.0955	96.16	87.5	[84.9 - 89.7%	0.0149	94.86	90.1	[87.3 - 92.3%]	0.109	99.43	0.04
FW306-06-A	100	[100 - NaN%]	0	99.62	100	[100 - NaN%]	0	98.3	100	[100 - NaN%]	0	99.99	0

Query genome: FW306-07-I

	Formula 1				Formula 2				Formula 3				G+C difference
Reference genome	DDH	Model C.I.	Distance	Prob. DDH >= 70%	DDH	Model C.I.	Distance	Prob. DDH >= 70%	DDH	Model C.I.	Distance	Prob. DDH >= 70%	
FW305-123	18.2	[15.1 - 21.7%]	0.7648	0	20.9	[18.6 - 23.3%]	0.2105	0	17.8	[15.2 - 20.8%]	0.8143	0	3.49
FW305-BF8	23.8	[20.5 - 27.4%]	0.6227	0.01	22.6	[20.3 - 25%]	0.1941	0	22.5	[19.7 - 25.6%]	0.6959	0	0.31
FW306-2-2C-D06B	18.2	[15.1 - 21.8%]	0.7633	0	21.5	[19.3 - 24%]	0.2037	0	17.9	[15.3 - 20.9%]	0.8115	0	1.96
FW306-04-A	18.1	[15.1 - 21.7%]	0.7655	0	21.5	[19.2 - 23.9%]	0.2045	0	17.8	[15.2 - 20.8%]	0.8135	0	2.21
FW306-05-C	90.5	[87.3 - 92.9%]	0.0782	97.46	90	[87.6 - 91.9%]	0.0122	95.78	92.9	[90.6 - 94.7%]	0.0894	99.71	0.04
FW306-06-A	87.9	[84.5 - 90.7%]	0.0919	96.47	87.9	[85.4 - 90%]	0.0144	95.02	90.7	[88 - 92.8%]	0.105	99.5	0.08
FW306-07-I	100	[100 - NaN%]	0	99.62	100	[100 - NaN%]	0	98.3	100	[100 - NaN%]	0	99.99	0

Table S3. Genes found in the predicted genomic islands for each of the seven isolates and the start and end locations of each gene in the genome (bp). Genes annotated as hypothetical proteins were not included in this table.

**Gene product**	**Gene start**	**Gene end**
**FW305-123**		
Chloramphenicol efflux pump	4778823	4780049
D-inositol-3-phosphate glycosyltransferase	4783223	4784314
HTH-type transcriptional repressor GlcR	7409738	7410529
3-oxo-isoapionate kinase	7411530	7412831
Putative KHG/KDPG aldolase	7413832	7414473
ATP-dependent Clp protease ATP-binding subunit ClpL	7417374	7419140
Dihydroanticapsin 7-dehydrogenase	7420141	7420890
Adaptive-response sensory-kinase SasA	7421891	7423597
Glucosamine-6-phosphate deaminase	7429178	7429969
Putative glyoxylase CFP32	7435422	7436204
Bacitracin transport ATP-binding protein BcrA	7438742	7439452
Arabinose metabolism transcriptional repressor	7443764	7444912
Alpha-ketoglutarate permease	7445913	7447214
Hydrogen cyanide synthase subunit HcnC	7449560	7450828
Fosfomycin resistance protein AbaF	7453363	7454706
Ribosome maturation factor RimP	7459894	7460505
HTH-type transcriptional repressor YtrA	7801190	7801579
HTH-type transcriptional regulator SutR	8419642	8420241
putative acyl--CoA ligase YhfT	8421242	8422633
HTH-type transcriptional regulator DegA	8428382	8429434
2-succinyl-6-hydroxy-2, 4-cyclohexadiene-1-carboxylate synthase	8430435	8431274
Gluconolactonase	8434166	8435047
**FW305-BF8**		
HTH-type transcriptional regulator KipR	3024922	3025674
IS30 family transposase ISCmi3	3479258	3479794
Hypoxic response protein 1	3482053	3482469
Peptidyl-tRNA hydrolase	3484803	3485387
RNA polymerase-binding transcription factor DksA	4113559	4113912
Muconolactone Delta-isomerase	4116234	4116512
Deacetylase	4118852	4120042
D-methionine transport system permease protein MetI	4122553	4123224
Melibiose/raffinose/stachyose import permease protein MelC	4124225	4125115
Fosfomycin resistance protein AbaF	4126116	4127477

Bifunctional protein FolD protein	4128478	4129356
Aspartate-semialdehyde dehydrogenase	4130357	4131499
D-inositol 3-phosphate glycosyltransferase	4132500	4133756
Tyrosine recombinase XerC	4134757	4135629
PEP-dependent dihydroxyacetone kinase, ADP-binding subunit DhaL	4136630	4137256
Phosphatidylinositol phosphate synthase	4145318	4145938
NAD-dependent glycerol dehydrogenase	4146939	4147685
NADH pyrophosphatase	4152021	4153073
D-alanine aminotransferase	4154074	4155006
Sorbitol dehydrogenase	4157727	4158806
Ammonia channel	4159807	4161177
Cell division protein FtsQ	4165867	4167030
Molybdenum import ATP-binding protein ModC	4169961	4171010
Cold shock protein	4173380	4173583
Transcriptional regulatory protein YpdB	4177517	4178236
Multidrug resistance protein MdtH	4179237	4180430
Putative multidrug resistance protein MdtD	4184559	4185950
Gluconate 5-dehydrogenase	4193173	4193943
Putative fluoride ion transporter CrcB	4194944	4195309
Leucine efflux protein	4197778	4198413
Putative agmatine deiminase	4199414	4200388
L-threonine 3-dehydrogenase	5279857	5280864
IS21 family transposase IS1415	5281865	5282650
Alkyl hydroperoxide reductase E	5286632	5287135
Serine hydroxymethyltransferase 2	5288136	5289476
Thiol:disulfide interchange protein DsbD	5290477	5291226
Protease PrtS	5294073	5295113
putative multidrug resistance protein EmrY	5296114	5297625
putative sugar epimerase YhfK	5298626	5299297
3-oxoacyl-[acyl-carrier-protein] reductase FabG	5306253	5307029
Transcriptional regulatory protein CitT	5308030	5308698
Vitamin B12 import ATP-binding protein BtuD	5314018	5314944
putative transcriptional regulatory protein TcrX	5323714	5324454
DNA ligase C	8004087	8005154
Maltose O-acetyltransferase	8006155	8006733
Glutathione-independent formaldehyde dehydrogenase	8011946	8013094
IS481 family transposase ISArsp1	8015353	8016330
putative trans-aconitate 2-methyltransferase	8017331	8018104
putative protein YyaP	8904047	8904646

NADPH dehydrogenase	8907433	8909568
Molybdate-binding protein ModA	8910569	8911372
Na(+)/H(+) antiporter NhaA	8922848	8924200
**FW306-2-2C-D06B**		
Phage shock protein A	966796	967584
putative oxidoreductase/MSMEI_2347	968585	969430
putative enoyl-CoA hydratase echA8	970431	971210
putative 3-hydroxyisobutyrate dehydrogenase	972211	973131
Acyl-CoA dehydrogenase	974132	975268
Electron transfer flavoprotein subunit beta	978202	978969
Catabolite control protein A	3300451	3301506
Ferredoxin--NADP reductase	4392419	4394137
Hydroxyacylglutathione hydrolase	4395138	4396196
Putative aminoacrylate hydrolase RutD	4397197	4398063
Acyl-coenzyme A thioesterase PaaI	4404813	4405295
HTH-type transcriptional regulator DegA	4406296	4407357
Leucine-responsive regulatory protein	4408358	4408810
Nitric oxide reductase FlRd-NAD(+) reductase	4409811	4410908
Bifunctional riboflavin kinase/FMN adenylyltransferase	4411909	4412892
Putative 4-hydroxy-4-methyl-2-oxoglutarate aldolase	4641783	4642265
Urocanate hydratase	4643266	4644993
Multidrug efflux system permease protein	5453995	5455617
Protoporphyrinogen oxidase	6272990	6274459
p-aminobenzoyl-glutamate hydrolase subunit B	6276580	6278199
IS481 family transposase ISMysp5	6279200	6280198
Diacetylchitobiose uptake system permease protein NgcG	6284537	6285475
**FW306-04-A**		
Guanine deaminase	2456826	2457311
Cold shock protein	2468819	2469022
3-methyl-2-oxobutanoate dehydrogenase subunit beta	2665026	2666060
Oxygen sensor histidine kinase response regulator DevS/DosS	2667061	2668779
Nitrite reductase (NADH) small subunit	2677936	2678307
Undecaprenyl-diphosphatase	2680530	2681399
Ribokinase	2686436	2687542
Biotin carboxylase	2688543	2689895
Chaperone protein DnaJ	4377544	4378065
ATP-dependent DNA helicase Rep	4380717	4383491
Iron-sulfur cluster carrier protein	5019352	5020551
IS30 family transposase ISPfr16	5919310	5920491

Uric acid degradation bifunctional protein	5923443	5923970
Putative multidrug resistance protein MdtD	5926985	5929024
Thiol:disulfide interchange protein DsbD	5931826	5932581
Alanine dehydrogenase	5934915	5936033
Multidrug resistance protein MdtH	6422036	6423076
Exodeoxyribonuclease 7 small subunit	6428970	6429212
**FW306-05-C**		
Tyrosine recombinase XerC	3750475	3752460
Putative glyoxylase CFP32	3753461	3754234
Universal stress protein	3757684	3758136
NADP-dependent fatty aldehyde dehydrogenase	3771872	3773323
Vanillate/3-O-methylgallate O-demethylase	3777578	3778990
Cobalt/magnesium transport protein CorA	3779991	3781007
D-allose transport system permease protein AlsC	3783197	3784192
Transcriptional regulatory protein LiaR	3785193	3785819
IS110 family transposase IS117	3790200	3791432
Flavin-dependent monooxygenase, reductase subunit HsaB	3796978	3797493
Tryptophan-rich protein TspO	3798494	3799015
HTH-type transcriptional repressor RspR	3800016	3800672
Lipoprotein LipO	3804462	3805994
Glycerol-3-phosphate regulon repressor	3810168	3810956
Multidrug efflux system permease protein	3817801	3819423
Formate dehydrogenase subunit alpha	3820424	3820990
Malate synthase	4054777	4056375
ATP synthase subunit delta	4059027	4059854
Intracellular exo-alpha-(1->5)-L-arabinofuranosidase	4060855	4062381
IS110 family transposase IS117	4071421	4072653
High-affinity branched-chain amino acid transport ATP-binding protein LivF	4076770	4077480
Trehalose transport system permease protein SugA	4084965	4085900
Putative oxidoreductase CatD	4089930	4090364
Potassium-transporting ATPase ATP-binding subunit	4239845	4242439
Hydroxyacylglutathione hydrolase	4248170	4248994
Intracellular exo-alpha-(1->5)-L-arabinofuranosidase	4253261	4254799
UDP-N-acetylglucosamine 4-epimerase	4255980	4256729
IS3 family transposase ISMsm7	4257730	4258017
O-acetyl-L-homoserine sulfhydrylase	4266736	4268082
FMNH(2)-dependent dimethylsulfone monooxygenase	5046703	5047950
Penicillin-binding protein 1A	5421544	5423763
putative methyltransferase	5424764	5425522

Ribosomal silencing factor RsfS	5432922	5433323
Nitrite reductase (NADH) small subunit	5435591	5435974
IS3 family transposase ISMsm7	5438500	5438787
KHG/KDPG aldolase	5439794	5440456
UDP-3-O-(3-hydroxymyristoyl)glucosamine N-acyltransferase	5441457	5442104
Guanidinium exporter	5443105	5443428
Putative cardiolipin synthase	5446110	5446733
3-oxoacyl-[acyl-carrier-protein] synthase 3	5451984	5452967
Mercuric resistance operon regulatory protein	5739260	5739628
23S rRNA (guanosine-2'-O-)-methyltransferase RlmB	5740629	5741438
Dihydroanticapsin 7-dehydrogenase	5742439	5743203
putative protein YihR	5746881	5747783
putative trans-aconitate 2-methyltransferase	5748784	5749569
5-formyltetrahydrofolate cyclo-ligase	5751873	5752493
Regulatory protein RecX	5753494	5754069
IS3 family transposase ISAar26	6473359	6474243
Homoserine/homoserine lactone efflux protein	6476763	6477386
2-keto-3-deoxygluconate permease	6478387	6479376
Enterobactin exporter EntS	6488105	6489376
5-amino-6-(5-phospho-D-ribitylamino)uracil phosphatase YitU	6643612	6644475
Pyruvate dehydrogenase E1 component	6648280	6651048
IS3 family transposase ISMsm7	6656974	6657261
FAD:protein FMN transferase	6957303	6958241
Putative pyridoxal phosphate-dependent aminotransferase EpsN	6959242	6960378
HTH-type transcriptional repressor NagR	7270458	7271198
Shikimate kinase	7286135	7286704
IS3 family transposase ISBli34	7290322	7291134
Metal cation efflux system protein CzcD	7296775	7297692
Peptide deformylase	7749628	7750230
HTH-type transcriptional regulator CdhR	7763778	7764782
3-phenylpropionate-dihydrodiol/cinnamic acid-dihydrodiol dehydrogenase	7765783	7766637
ATP-dependent DNA helicase Rep	7769343	7772006
Holliday junction ATP-dependent DNA helicase RuvA	7773007	7773651
putative deferrochelatase/peroxidase EfeN	7783273	7784652
Glycogen synthase	7785653	7786666
Tyrosine recombinase XerC	7849788	7850714
ABC transporter glutamine-binding protein GlnH	7851715	7852602
putative alcohol dehydrogenase AdhA	7867991	7869010
Zinc-dependent sulfurtransferase SufU	7872650	7873099

HTH-type transcriptional regulator DegA	8007452	8008456
Tn3 family transposase ISNpu13	8023621	8026587
putative flavin reductase	8037440	8037940
Multiple sugar-binding protein	8056116	8057402
UDP-N-acetyl-D-glucosamine 6-dehydrogenase	8437563	8438831
**FW306-06-A**		
NAD(P)H-quinone oxidoreductase subunit 3	2559064	2559414
Tyrosine recombinase XerC	2560415	2561542
putative cation-transporting ATPase G	3430649	3432601
D-galactonate dehydratase family member RspA	3433602	3434831
Aldose sugar dehydrogenase YliI	3435832	3436788
Putative two-component membrane permease complex subunit SMU_747c	3441225	3442103
putative sugar epimerase YhfK	3448672	3449328
Multidrug resistance protein Stp	3462621	3464066
18 kDa heat shock protein	3465067	3465492
Putative glutamate--cysteine ligase 2	3467901	3469046
Thymidine kinase	3470047	3470775
Putative transport protein	3473871	3475004
D-galactonate dehydratase	3476005	3477213
3D-(3,5/4)-trihydroxycyclohexane-1,2-dione hydrolase	3480051	3482027
Urease accessory protein UreD	3483028	3483777
Dihydrolipoyl dehydrogenase	3503603	3505057
HTH-type transcriptional regulator LutR	3506058	3506762
Glucitol operon repressor	3507763	3508536
ECF RNA polymerase sigma factor SigL	3513779	3514279
Phosphoenolpyruvate synthase	3520340	3522769
HTH-type transcriptional regulator XynR	3527195	3527947
Transcriptional regulator SlyA	4054902	4055459
Glycine betaine-binding protein YehZ	4060798	4061760
3-carboxy-cis,cis-muconate cycloisomerase	4066738	4068210
DNA-invertase hin	4079685	4080338
Flavohemoprotein	4082609	4084012
Thiol-disulfide oxidoreductase ResA	4100573	4101184
Transcriptional regulatory protein TcrA	4939683	4940366
Iron-sulfur cluster carrier protein	4942931	4944478
IS256 family transposase ISMlu1	5085082	5086329
IS481 family transposase ISPfr21	5087330	5088316
putative cation efflux system protein	5092613	5093671

3-keto-5-aminohexanoate cleavage enzyme	5099355	5100287
putative HTH-type transcriptional regulator YybR	5101288	5101662
Enterobactin exporter EntS	5105332	5106687
Putrescine transporter PotE	5107688	5109190
Organic hydroperoxide resistance transcriptional regulator	5122344	5122778
ATP synthase subunit b	5219802	5220353
HTH-type transcriptional regulator LutR	5231034	5231732
IS21 family transposase ISLxx3	5236047	5236865
Pyrimidine-specific ribonucleoside hydrolase RihA	5237866	5238891
NH(3)-dependent NAD(+) synthetase	5241282	5242157
Ribosomal silencing factor RsfS	5254510	5254911
Nicotine blue oxidoreductase	5257902	5258696
L-lactate transporter	7124753	7126138
Putative ABC transporter substrate-binding protein YesO	7133527	7134816
Succinate-semialdehyde dehydrogenase	7137324	7138823
Chromosome partition protein Smc	7145407	7147740
Tagatose-6-phosphate kinase	7148741	7149763
tRNA (guanine-N(7)-)-methyltransferase	7152100	7153023
IS21 family transposase ISBmu3	7155321	7156088
RNA polymerase-associated protein RapA	7243201	7247502
RNA polymerase-binding transcription factor DksA	7512025	7512384
putative oxidoreductase YohF	7976725	7977522
High-affinity branched-chain amino acid transport ATP-binding protein LivF	7987888	7988598
ATP-dependent RNA helicase SrmB	8391885	8396549
**FW306-07-I**		
IS481 family transposase ISAar35	3600490	3601458
Metal cation efflux system protein CzcD	3602459	3603376
Pca regulon regulatory protein	3604377	3605186
Hydroxyacylglutathione hydrolase GloC	3606187	3606837
Glycine cleavage system H protein	3607838	3608221
Transcriptional regulator SlyA	3611332	3611862
Membrane-bound lytic murein transglycosylase C	5263523	5264902
Proofreading thioesterase EntH	5275262	5275726
IS30 family transposase ISMsm8	5317013	5318410
Fatty acid ABC transporter ATP-binding/permease protein	5322293	5322934
D-tagatose-1,6-bisphosphate aldolase subunit GatY	5323935	5324588
Bifunctional NAD(P)H-hydrate repair enzyme Nnr	5326946	5327905
NADPH oxidoreductase	5338273	5339370
phosphoglycerate mutase GpmB	5340371	5340955

2-succinyl-6-hydroxy-2, 4-cyclohexadiene-1-carboxylate synthase	5341956	5344364
2-keto-3-deoxy-L-rhamnonate aldolase	5346935	5347708
Sensor histidine kinase DcuS	5353439	5355181
N-(5'-phosphoribosyl)anthranilate isomerase	5357500	5358111
Tyrosine recombinase XerC	5507070	5509340
Glucitol operon repressor	5514826	5515599
Multidrug efflux system ATP-binding protein	5519404	5520402
Putative pre-16S rRNA nuclease	6377842	6378441
N5-carboxyaminoimidazole ribonucleotide mutase	6391185	6391766
Tetracycline repressor protein class B from transposon Tn10	6392767	6393471
Thiosulfate sulfurtransferase GlpE	6400214	6400555
2,3-bisphosphoglycerate-dependent phosphoglycerate mutase	6983147	6983938
Methionine aminopeptidase 1	6986956	6987729
Sulfurtransferase	6988730	6989026
putative amino acid permease YhdG	6995977	6997503
IS110 family transposase ISAar41	6999708	7000757
Heat shock protein 15	7085497	7085880
Oxidoreductase YdhF	7086881	7087822
lipid kinase YegS	7088823	7089902
IS481 family transposase ISAar35	7092527	7093495

Table S4. Results of the carbon utilization assay for all seven isolates. OD_{600} values are the mean of three biological replicates. The color scale correlates with OD_{600} from low (white) to high (red). Carbon sources are grouped by compound type.

	123	BF8	D06B	04-A	05-C	06-A	07-I
cytosine	0.14	0.07	0.11	0.20	0.08	0.10	0.08
thymidine	0.12	0.12	0.19	0.12	0.16	0.10	0.09
inosine	0.65	0.22	0.35	0.11	0.08	0.09	0.08
cytodine	0.26	0.08	0.33	0.14	0.08	0.09	0.08
uridine	0.67	0.16	0.28	0.31	0.12	0.17	0.08
adenosine	0.65	0.14	0.24	0.13	0.09	0.13	0.14
adenine	0.11	0.17	0.18	0.11	0.10	0.16	0.08
tryptophan	0.14	0.20	0.22	0.13	0.13	0.19	0.10
arginine	0.53	0.14	0.19	0.22	0.23	0.27	0.23
asparagine	0.55	0.18	0.31	0.31	0.30	0.33	0.38
aspartate	0.16	0.08	0.12	0.22	0.08	0.13	0.10
glutamate	0.28	0.10	0.20	0.23	0.09	0.11	0.14
histidine	0.76	0.16	0.42	0.42	0.26	0.40	0.55
glutamine	0.66	0.18	0.36	0.42	0.32	0.30	0.44
cysteine	0.13	0.08	0.15	0.11	0.13	0.12	0.09
tyrosine	0.11	0.08	0.42	0.30	0.48	0.39	0.08
serine	0.28	0.08	0.12	0.15	0.09	0.16	0.11
proline	0.22	0.15	0.20	0.27	0.18	0.21	0.25
isoleucine	0.11	0.07	0.12	0.10	0.08	0.10	0.08
methionine	0.12	0.16	0.17	0.11	0.16	0.14	0.07
phenylalanine	0.13	0.17	0.20	0.11	0.10	0.14	0.08
citrulline	0.12	0.16	0.36	0.11	0.10	0.17	0.08
glucosamine	0.41	0.25	0.46	0.41	0.35	0.37	0.39
casamino acids	0.47	0.30	0.50	0.40	0.32	0.49	0.40
xylitol	0.11	0.14	0.15	0.14	0.08	0.10	0.08
mannitol	0.11	0.29	0.67	0.10	0.43	0.54	0.58
ducitol	0.11	0.07	0.10	0.17	0.09	0.22	0.09
myo-inositol	0.12	0.12	0.17	0.10	0.09	0.20	0.10
acetate	0.25	0.12	0.17	0.14	0.20	0.21	0.21
malate	0.49	0.19	0.23	0.26	0.33	0.39	0.29
folate	0.23	0.17	0.67	0.66	0.72	0.69	0.38
succinate	0.74	0.19	0.35	0.25	0.27	0.34	0.26
propionate	0.12	0.11	0.16	0.16	0.21	0.25	0.20
formate	0.12	0.15	0.19	0.11	0.08	0.15	0.08
butyrate	0.12	0.08	0.16	0.23	0.14	0.22	0.22
fumarate	0.20	0.18	0.19	0.11	0.12	0.16	0.12
citrate	0.24	0.21	0.31	0.19	0.25	0.41	0.31
lactate	0.35	0.19	0.23	0.13	0.22	0.39	0.39
pyruvate	0.37	0.21	0.28	0.16	0.30	0.42	0.35
glycolic acid	0.11	0.16	0.16	0.11	0.15	0.16	0.08
nicotinic acid	0.12	0.10	0.09	0.11	0.08	0.10	0.08
pthalate	0.12	0.08	0.10	0.11	0.08	0.10	0.08
sucrose	0.12	0.36	0.75	0.61	0.50	0.59	0.50
glucose	0.86	0.46	0.68	0.57	0.48	0.63	0.54
lactose	0.18	0.32	0.39	0.30	0.24	0.19	0.18
ribose	0.14	0.18	0.18	0.14	0.18	0.17	0.24
mannose	0.32	0.27	0.23	0.12	0.21	0.32	0.28
fructose	0.97	0.37	0.65	0.53	0.59	0.65	0.59
rhamnose	0.13	0.15	0.16	0.11	0.09	0.14	0.09
maltose	0.34	0.21	0.30	0.12	0.52	0.42	0.40
arabinose	0.17	0.10	0.11	0.11	0.11	0.25	0.20
cellobiose	0.52	0.26	0.30	0.12	0.35	0.48	0.34
cellulose	0.11	0.10	0.10	0.11	0.08	0.10	0.08
glucuronamide	0.27	0.21	0.26	0.25	0.20	0.27	0.20
urea	0.12	0.08	0.10	0.11	0.08	0.10	0.08
tryptone	0.47	0.17	0.22	0.14	0.32	0.32	0.17
R2A	0.68	0.37	0.60	0.53	0.51	0.54	0.52
TSB	0.98	0.48	0.75	0.70	0.56	0.68	0.60
no C source	0.12	0.08	0.09	0.10	0.09	0.09	0.08
no cells	0.09	0.07	0.09	0.08	0.08	0.09	0.09

Table S5. Predicted genomic capability to catabolism 62 simple carbon substrates of the seven isolates presented in this study, listed alphabetically. A (+) indicates a high confidence prediction, (m) indicates a medium-confidence prediction, and (-) indicates a low confidence prediction, per default GapMind standards (Price et al., 2019).

	Groundwater		Sediment				
	123	BF8	D06B	04-A	05-C	06-A	07-I
acetate	+	+	+	+	+	+	+
D-alanine	-	-	-	-	-	-	-
alanine	+	+	+	m	+	+	+
arabinose	+	+	+	+	+	+	+
arginine	m	-	-	-	-	-	-
asparagine	+	+	+	+	+	+	+
aspartate	m	m	m	m	m	m	m
cellobiose	+	+	+	+	+	+	+
citrate	-	+	+	+	+	+	+
citrulline	-	-	-	-	-	-	-
deoxyinosine	-	-	-	-	-	-	-
deoxyribonate	-	-	-	-	-	+	-
deoxyribose	-	-	-	-	-	-	-
ethanol	+	m	+	+	m	m	m
fructose	m	m	m	m	m	m	m
fucose	-	-	-	-	-	-	-
fumarate	+	+	+	+	+	+	+
galactose	m	+	m	m	+	+	+
galacturonate	-	-	-	-	m	-	-
gluconate	+	m	+	+	-	m	m
glucose	+	+	+	+	+	+	+
glucose-6-P	-	-	-	-	-	-	-
glucosamine	-	-	-	-	-	-	-
glucuronate	-	-	-	-	-	-	-
glutamate	m	m	m	m	m	m	m
glycerol	m	m	m	m	m	m	m
histidine	m	m	m	+	m	m	m
isoleucine	-	-	m	-	m	m	m
4-hydroxybenzoate	-	-	-	-	-	-	-
D-lactate	m	m	m	-	m	m	m
L-lactate	+	+	m	+	+	+	+
lactose	-	-	-	-	-	-	-
leucine	-	-	-	-	m	m	m
lysine	-	m	-	-	-	-	-
L-malate	+	+	+	+	+	+	+
maltose	+	+	+	+	+	+	+
mannitol	+	+	+	+	+	+	+
mannose	m	m	-	-	m	m	m
myoinositol	-	-	-	-	-	-	-
NAG	-	-	-	-	-	-	-
2-oxoglutarate	+	+	m	+	+	+	+
phenylacetate	m	-	m	m	m	m	m
phenylalanine	m	-	m	m	m	m	m
proline	-	-	-	-	m	-	-
propionate	+	+	+	+	+	+	+
putrescine	m	m	m	m	-	m	m
pyruvate	+	+	+	+	+	+	+
rhamnose	-	m	m	-	-	-	-
ribose	+	m	-	-	-	-	-
D-serine	-	-	-	-	-	-	-
serine	m	m	-	-	-	-	-
sorbitol	-	m	-	m	-	m	m
succinate	+	+	+	+	+	+	+
sucrose	+	+	+	+	+	+	+
threonine	m	m	m	m	m	m	m
thymidine	-	-	-	-	-	-	-
trehalose	m	m	m	m	m	m	m
tryptophan	-	-	-	-	-	-	-
tyrosine	-	-	-	-	-	-	-
valine	-	-	m	-	-	-	-
xylitol	-	-	-	-	-	-	-
xylose	m	+	+	m	+	+	+

Table S6. 34 Publicly available *Arthrobacter* included in the pangenome analysis. For any publicly available isolate without a publication reference, environment of origin was obtained from the NCBI metadata associated with the given accession number.

No.	Strain	Accession Number	Environment of Origin	Publication Reference
1	Arthrobacter nitroguajacolicus Rue61a	GCA_000294695	Wastewater	(Niewerth et al., 2012)
2	Arthrobacter alpinus R3.8	GCA_001294625.1	Permafrost	(See-Too et al., 2017)
3	Arthrobacter alpinus A3	GCA_000755585.2	Permafrost	
4	Arthrobacter alpinus ERGS4:06	GCA_001445575.1	Freshwater	(Kumar et al., 2016)
5	Arthrobacter sp. ERGS1:01	GCA_001281315.1	Environmental	
6	Arthrobacter sp. ATCC 21022	GCA_001542565.1	Soil	(Russell and Hatfull, 2016)
7	Arthrobacter sp. ZXY-2	GCA_001854365.1	Soil	
8	Arthrobacter sp. U41	GCA_001750145.1	Freshwater	(Fomenkov et al., 2017)
9	Arthrobacter sp. Hiyo8	GCA_001549895.1	Soil	(Hiraoka et al., 2016)
10	Arthrobacter sp. QXT-31	GCA_001969265.1	Soil	
11	Arthrobacter sp. YN	GCA_002224285.1	Environmental	
12	Arthrobacter sp. Hiyo4	GCA_002370555.1	Soil	(Hiraoka et al., 2016)
13	Arthrobacter crystallopoietes DSM 20117	GCA_002849715.1	Soil	
14	Arthrobacter agilis UMCV2	GCA_002953615.1	Rhizosphere	
15	Arthrobacter sp. PAMC25564	GCA_004798705.1	Soil	(Han et al., 2021)
16	Arthrobacter dokdonellae DCT5	GCA_003268655.1	Environmental	(Koh et al., 2019)
17	Arthrobacter sp. MN0502	GCA_004001285.1	Desert soil	
18	Arthrobacter sp. KBS0702	GCA_005937985.2	Soil	
19	Arthrobacter sp. UKPF54-2	GCA_007858535.1	Soil	
20	Arthrobacter sp. TES	GCA_014863565.1	Soil	
21	Arthrobacter sp. PAMC 25486	GCA_000785535.1	Arctic soil	
22	Arthrobacter sp. PGP41	GCA_002953935.1	Rhizosphere	(Xu et al., 2018)
23	Arthrobacter sp. 24S4-2	GCA_005280255.1	Environmental	
24	Arthrobacter sp. AQ5-05	GCA_009707285.1	Arctic soil	(Lee et al., 2018)
25	Arthrobacter sp. D5-1	GCA_017357425.1	Soil	(Bazhanov et al., 2016)
26	Arthrobacter crystallopoietes NT16	GCA_017603825.1	Environmental	(Fan et al. 2019)
27	Arthrobacter sp. FB24	GCA_000196235.1	Soil	(Nakatsu et al., 2013)
28	Arthrobacter sp. NicSoilB11	GCA_019977375.1	Soil	(Shimasaki et al., 2021)
29	Arthrobacter sp. NicSoilB8	GCA_019977355.1	Soil	(Shimasaki et al., 2021)
30	Arthrobacter sp. NtRootA9	GCA_019977115.1	Endosphere	(Shimasaki et al., 2021)
31	Arthrobacter sp. NicSoilC5	GCA_019977395.1	Soil	(Shimasaki et al., 2021)
32	Arthrobacter sp. NicSoilB4	GCA_019977335.1	Soil	(Shimasaki et al., 2021)
33	Arthrobacter sp. NicSoilE8	GCA_019977415.1	Soil	(Shimasaki et al., 2021)
34	Arthrobacter sp. NtRootA1	GCA_019976975.1	Endosphere	(Shimasaki et al., 2021)

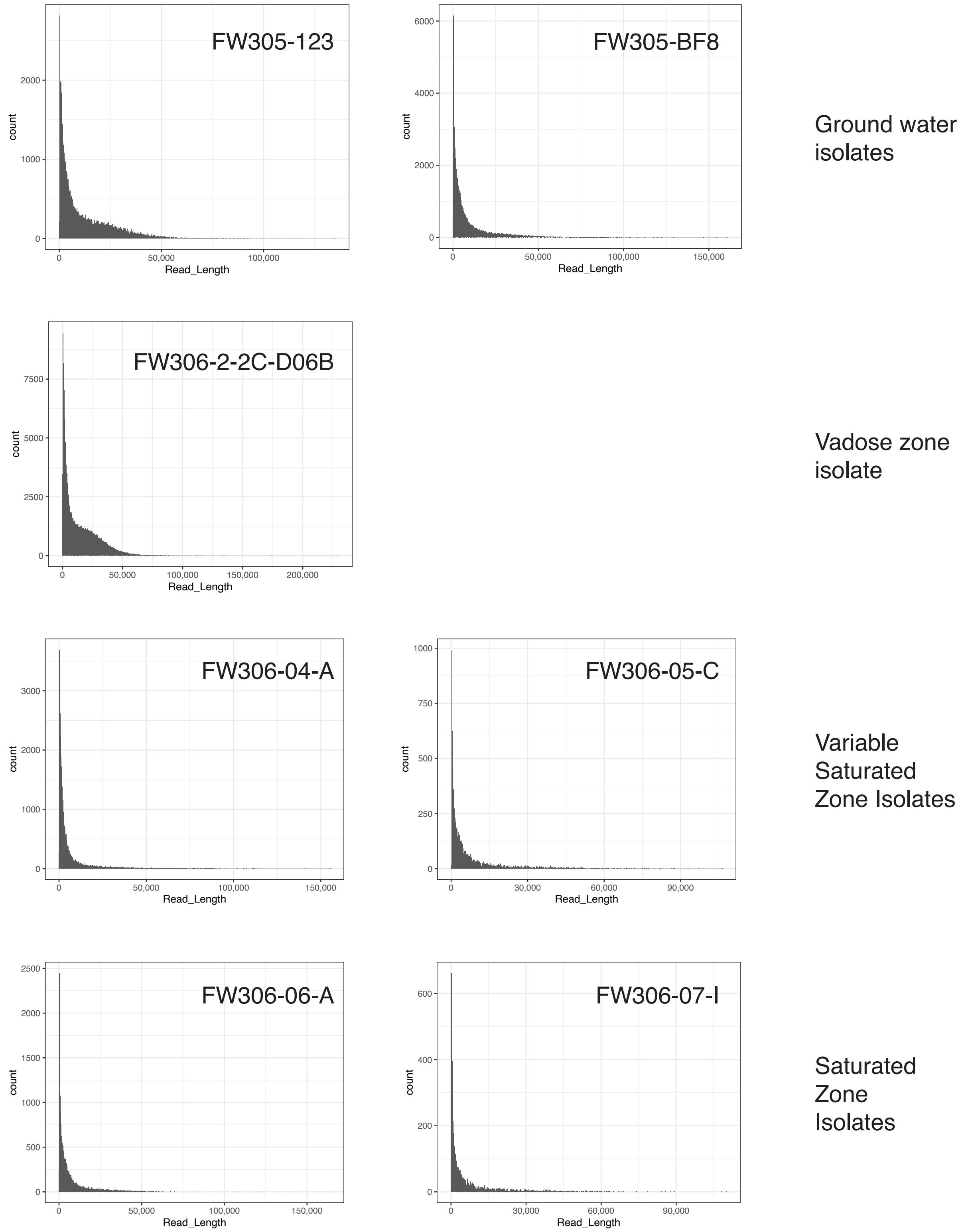

Figure S1. Long read distribution of Oxford Nanopore Technologies sequencing reads.

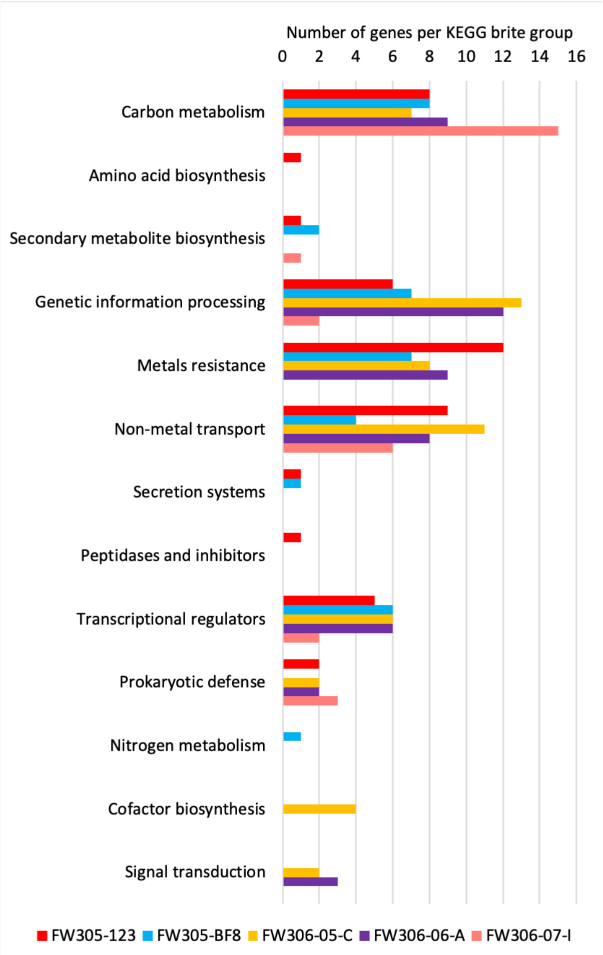

Figure S2. Protein-coding genes in plasmids of strains FW305-123, FW305-BF8, FW306-05-C, FW306-06-A, and FW306-07-I organized by KEGG brite group. Strains FW306-2-2C-D06B and FW306-04-A do not possess plasmids and are therefore omitted. Genes not mapping to a KEGG brite group are also omitted from this plot.

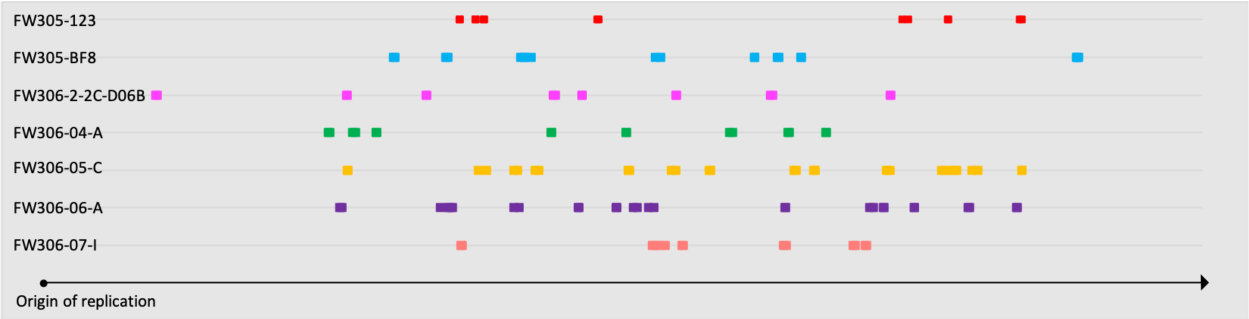

Figure S3. Predicted genomic islands in the seven *Arthrobacter* isolates mapped on their respective genomes beginning at the origin of replication (left). The genomes of groundwater strains 123 and BF8 contained 7 and 10 genomic islands, respectively. The genomes of sediment strains D06B, 04-A, 05-C, 06-A, and 07-I contained 8, 8, 14, 14, and 7 genomic islands, respectively.

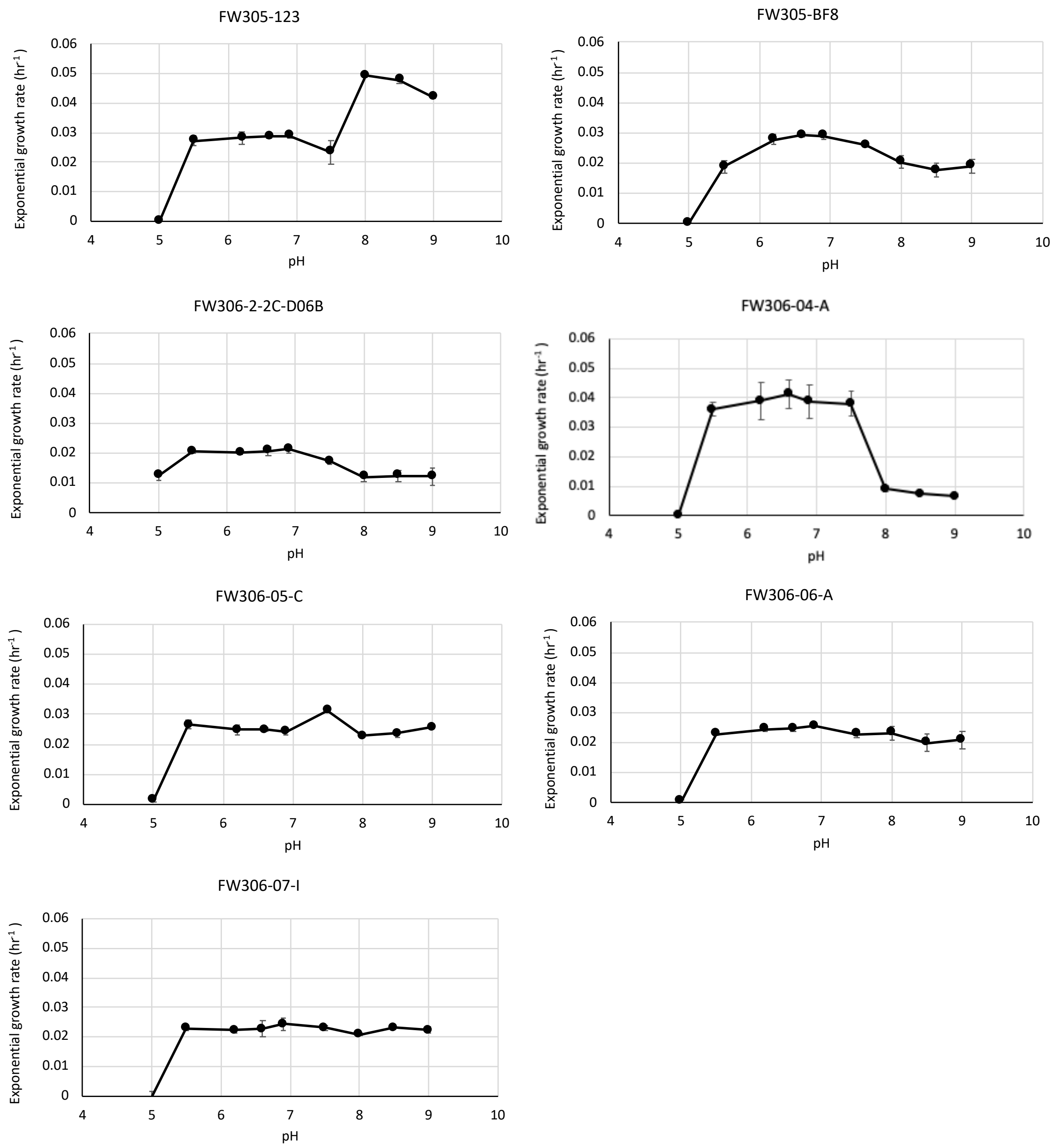

Figure S4. Exponential growth rates (in units of hr^{-1}) of isolates FW305-123, FW305-BF8, FW306-2-2C-D06B, FW306-04-A, FW306-05-C, FW306-06-A, and FW306-07-I over pH ranging from 5 to 9. Markers represent the mean of three biological replicates. Error bars indicate one standard deviation from the mean. Absence of error bars indicate the error is smaller than the marker.

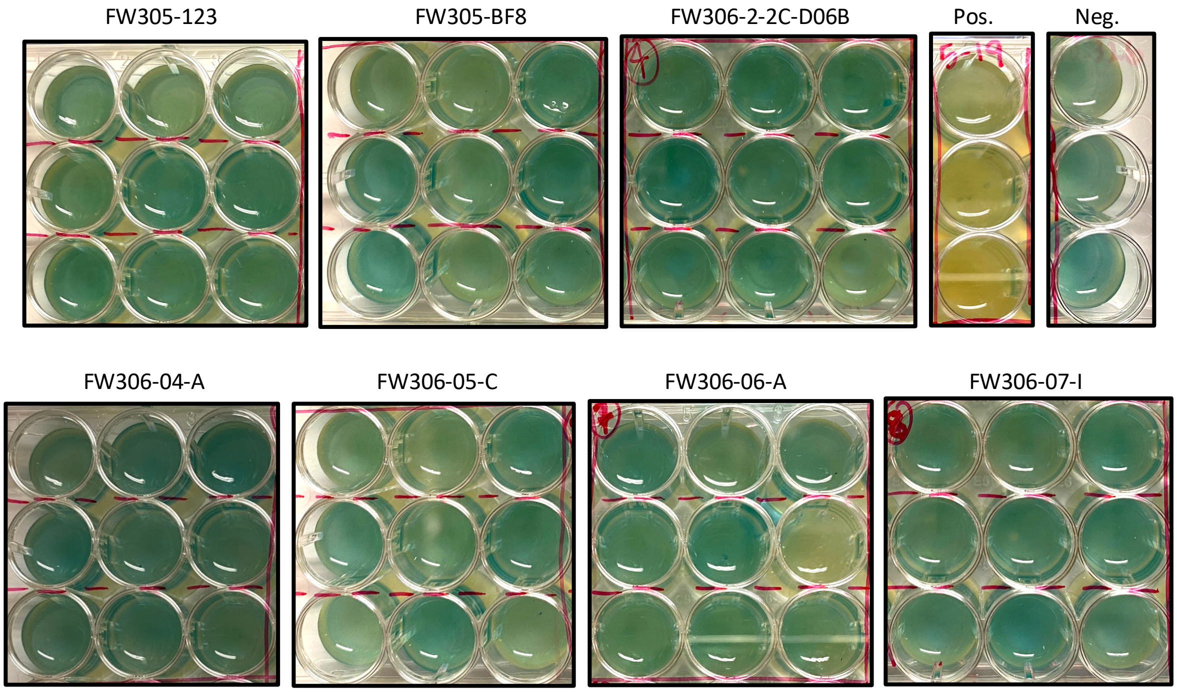

Figure S5. Images of colorimetric, overlay-chrome azurol S siderophore assay for the seven isolates (nine replicates) including positive control (Pos., three replicates) and negative control (Neg., three replicates). A resulting purple color indicated catechol-type siderophore production, yellow indicated hydroxamate-type siderophore production, red-orange indicated a mix of different siderophore types, and blue indicated the absence of detected siderophore production.

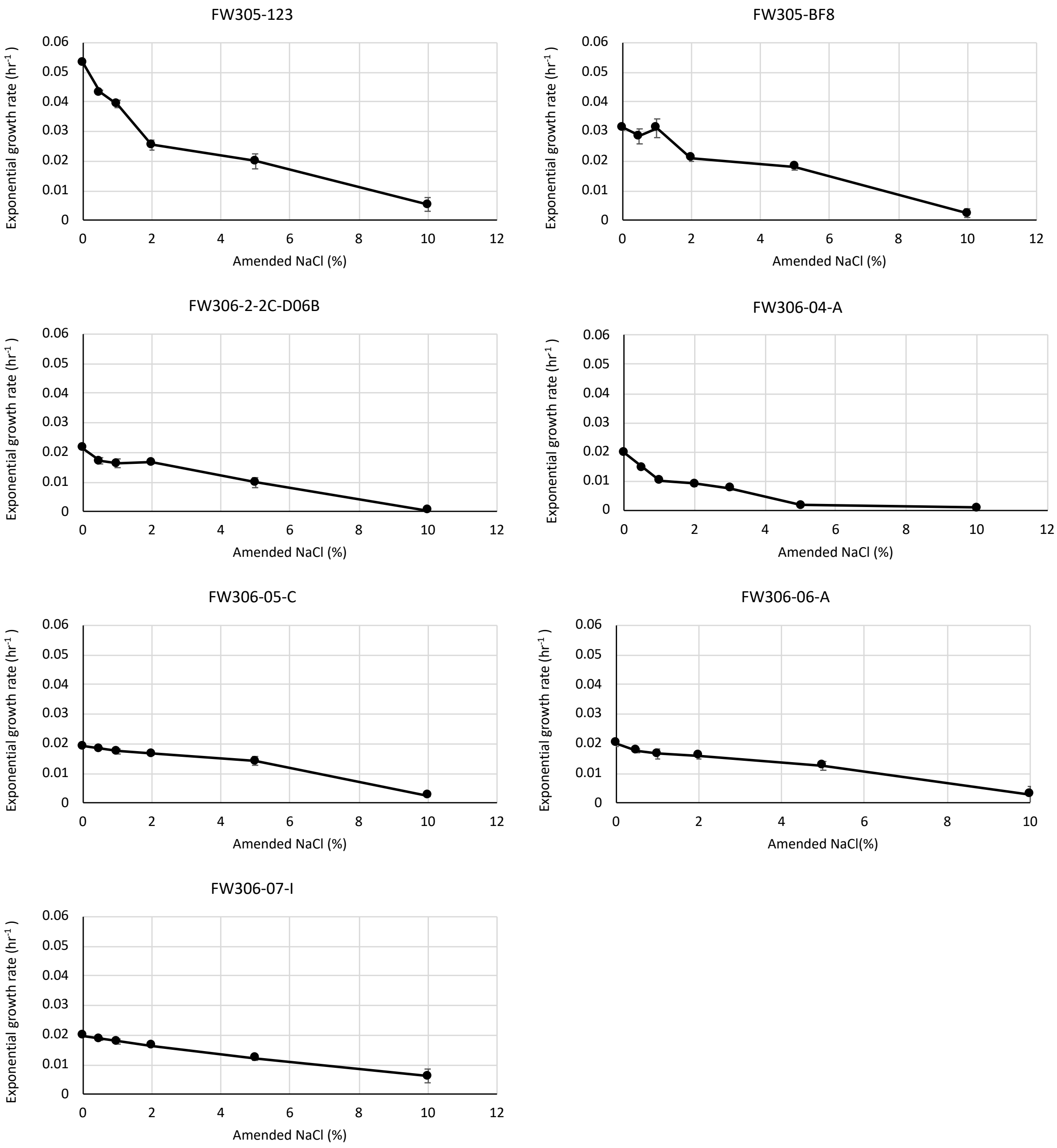

Figure S6. Exponential growth rates (in units of hr^{-1}) of isolates FW305-123, FW305-BF8, FW306-2-2C-D06B, FW306-05-C, FW306-06-A, and FW306-07-I over variable salinity ranging from 0.5% to 10% (w/v) amended to RCH2 mineral medium. Markers represent the mean of three biological replicates. Error bars indicate one standard deviation from the mean. Absence of error bars indicate the error is smaller than the marker.

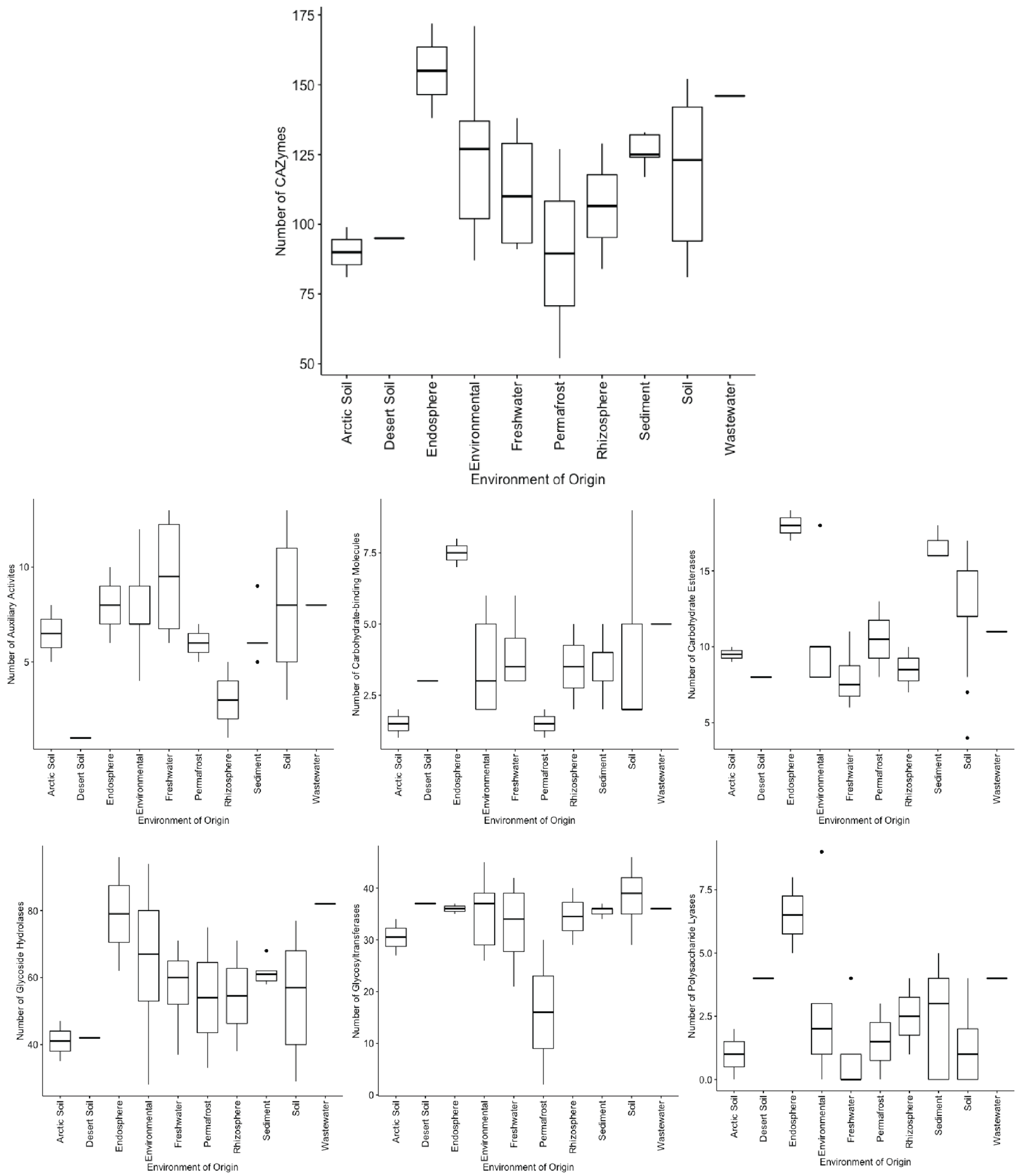

Figure S7. Box plots summarizing the predicted total CAZymes, auxiliary activities, carbohydrate-binding molecules, carbohydrate esterases, glycoside hydrolases, glycosyltransferases, and polysaccharide lyases by environment of origin. Differences in each category by environment of origin was evaluated via one-way ANOVA, with significant differences observed in carbohydrate esterases (p=0.00298) and glycosyltransferases (p=0.0237).

Supplemental Information File 2: Original 16S rRNA alignment of 41 *Arthrobacter* and 1 *Glutamicibacter* outgroup in fasta format.

Supplemental Information File 3: Trimmed 16S rRNA alignment of 41 *Arthrobacter* and 1 *Glutamicibacter* outgroup in fasta format.

Supplemental Information File 4: Phylogenetic tree of 41 *Arthrobacter* and 1 *Glutamicibacter* outgroup in Newick format.
